# Where do we stand with screening for colorectal cancer and advanced adenoma based on serum protein biomarkers? A systematic review

**DOI:** 10.1002/1878-0261.13734

**Published:** 2024-09-30

**Authors:** Adrien Grancher, Steven Cuissy, Hélène Girot, André Gillibert, Frédéric Di Fiore, Lydia Guittet

**Affiliations:** ^1^ U1086 “ANTICIPE” INSERM‐University of Caen Normandy, Centre François Baclesse Caen France; ^2^ Department of Hepato‐Gastroenterology and Digestive Oncology Rouen University Hospital France; ^3^ Department of Medical Biochemistry Rouen University Hospital France; ^4^ Department of Biostatistics Rouen University Hospital France; ^5^ Public Health Department Caen University Hospital France

**Keywords:** colorectal cancer, protein biomarkers, screening, serum

## Abstract

Colorectal cancer (CRC) screening has been proven to reduce both mortality and the incidence of this disease. Most CRC screening programs are based on fecal immunochemical tests (FITs), which have a low participation rate. Searching for blood protein biomarkers can lead to the development of a more accepted screening test. The aim of this systematic review was to compare the diagnostic potential of the most promising serum protein biomarkers. A systematic review based on PRISMA guidelines was conducted in the PubMed and Web of Science databases between January 2010 and December 2023. Studies assessing blood protein biomarkers for CRC screening were included. The sensitivity, specificity, and area under the ROC curve of each biomarker were collected. Among 4685 screened studies, 94 were considered for analysis. Most of them were case–control studies, leading to an overestimation of the performance of candidate biomarkers. The performance of no protein biomarker or combination of biomarkers appears to match that of the FIT. Studies with a suitable design and population, testing new assay techniques, or based on algorithms combining FIT with serum tests are needed.

## Graphical abstract

The search for a blood screening test for colorectal cancer is a major challenge. Many protein biomarkers have been identified as potentially interesting. However, their performance must be interpreted according to the design of the study. This systematic review reports protein biomarkers with the best potential for screening for colorectal cancer or advanced adenomas.

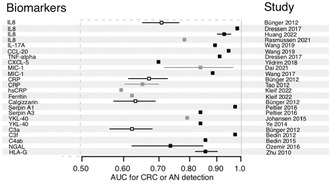

**Abbreviations**
AA: advanced adenoma
AN: advanced neoplasia
AUC: area under the ROC curve
CRC: colorectal cancer
ELISA: enzyme‐linked immunosorbent assay
FIT: fecal immunochemical test
HC: healthy control
NAA: non advanced adenoma
OS: overall survival

## 1 Introduction

Colorectal cancer (CRC) is a worldwide public health problem that affects 1.9 million people each year and causes 900 000 deaths [1]. Overall survival (OS) highly depends on the stage at diagnosis. The 5‐year survival rate is 92% for stage I patients, compared with 12% for stage IV patients [2]. CRC screening allows early detection at a preneoplastic stage or at a localized stage. Screen‐detected CRCs are more likely to be localized than nonscreen‐detected CRCs [3]. CRC screening programs have been proven to reduce both CRC‐specific mortality and CRC incidence [4]. Screening programs in several countries are based on noninvasive fecal occult blood tests. The fecal immunochemical test (FIT) offers good performance for CRC screening, with a sensitivity of 65–91% and specificity of 91–96% for CRC detection, varying according to the positivity threshold selected by the screening program [5, 6, 7, 8]. The global area under the ROC curve (AUC) of the FIT for CRC detection was estimated to be 0.95 (95% confidence interval [CI]: 0.93–0.97) [5]. However, participation rates remain low, under the European recommended threshold of 45% [9]. Among the explanatory factors, fecal aversion and the complexity of stool collection are important points [10]. Some data have suggested that a screening test based on blood sample collection would be more accepted by the general population than a stool‐based test [11, 12], or could be proposed in patients who declined colonoscopy and FIT [13]. Thus, developing a blood‐based screening test would increase the participation rate and therefore the effectiveness of screening.

Many blood biomarkers for CRC screening have been identified in the literature [14]. One blood‐based test detecting *SEPT9* methylation (Epi proColon®, Epigenomics AG, Berlin, Germany) is accredited by the FDA [15]. Recent results from the ECLIPSE clinical trial, evaluating a cell‐free DNA blood‐based test, have shown promising results [16]. However, these tests are still expensive and are not widely adopted as part of an organized screening program. Serum protein biomarkers could represent another promising option [14, 17]. Numerous studies have reported the use of several protein biomarkers for CRC and/or advanced adenoma (AA) detection. Nonetheless, most of these studies were retrospective studies with limited sample sizes that compared patients with invasive CRC to healthy controls (HCs). Few studies have included a study population that is representative of the population concerned by screening, on which the performance of biomarkers is to be evaluated [18]. Thus, studies including incident CRC cases diagnosed by screening colonoscopy in asymptomatic patients are to be preferred. Similarly, distinguishing a polyp or an adenoma group from a healthy control group is also an important criterion for assessing the specificity of biomarkers for CRC screening, but also for analyzing the performance of these biomarkers in detecting preneoplastic lesions [14, 17].

It is therefore necessary to sort through all this information to identify the most relevant serum protein biomarkers. The aim of this systematic review was to collect and compare the diagnostic ability of the most promising serum protein biomarkers for CRC screening.

## 2 Materials and Methods

This systematic review was conducted according to the Preferred Reporting Items for Systematic Review and Meta‐Analysis of Diagnostic Test Accuracy Studies (PRISMA‐DTA) guidelines for the literature search [19].

### 2.1 Search strategy

A full literature search of the PubMed and Web of Science databases was conducted on May 6, 2024. The following equation was used: (marker OR biomarker) AND (serum OR blood) AND (diagnosis OR screening) AND (colorectal OR colon OR rectal) AND (cancer OR carcinoma OR neoplasia). Additional terms were added to delete biomarkers of other cancers and genetic biomarkers: NOT (prostate OR lung OR breast OR gastric OR pancreatic OR ovarian OR thyroid) and NOT (DNA OR RNA OR microRNA).

One researcher (A.Gr) performed the data extraction. One reviewer (A.Gr) used the rayyan online software to delete duplicates and screen title and abstract of the extracted articles. The remaining articles were then assessed for eligibility in the full‐text review stage, blindly conducted by two reviewers (A.Gr and S.C.). In the event of disagreement between the two reviewers on the eligibility of an article, a collegial decision was made after rereading.

### 2.2 Study selection

Only articles in the English language published between January 1, 2010, and December 31, 2023, were considered. Animal or *in vitro* studies were excluded. Meta‐analyses and reviews without original data were excluded, as well as case report studies. Studies involving nonserum biomarkers (plasma, fecal, urine, saliva) were excluded. Studies reporting the ratio or index of interest between cells, or cells and proteins, were excluded. Studies involving genetic biomarkers (DNA, RNA, miRNA) were excluded. Studies focusing only on the prognostic value of biomarkers for CRC recurrence or metastasis were excluded, as well as the predictive value of treatment efficacy. Studies assessing serum protein biomarkers for CRC and/or colorectal adenoma diagnosis or screening were included. Studies that did not include the following three groups were excluded: a control group (with HC and/or other nonmalignant diseases), a polyp group (including adenomas), and a CRC group. Studies that did not specify the sensitivity, specificity, or AUC of the biomarkers were excluded.

### 2.3 Data collection process

Two researchers (A.Gr and S.C.) independently reviewed and collected the data from the included articles. Previous data on CRC carcinogenesis involving candidate biomarker(s), including population, study design, test(s) used to measure biomarker(s), diagnostic performance for CRC and/or adenoma detection, and comparisons with other well‐known biomarkers, have been systematically reported.

No sensitivity, specificity, or AUC calculations were performed, and only raw data from included studies were reported. Reported AUC, Sensitivity and Specificity of included studies for diagnosing CRC or advanced neoplasia (AN) were collected. When specified, the AUC, sensitivity, and specificity for adenoma or AA diagnosis were also collected. The AUC, sensitivity, and specificity for localized versus advanced CRC were also collected when available. AUC, sensitivity, or specificity of other standard biomarkers, such as CEA or CA19‐9 levels, were also collected if available. AUC, sensitivity, and specificity of other screening tests performed—such as fecal immunochemical test—were also collected if available.

### 2.4 Reporting quality and level of evidence

Two reviewers (A.Gr and S.C.) assessed the methodological quality of each included study using the Quality Assessment of Diagnostic Accuracy Studies 2 (QUADAS‐2) tool from the Cochrane Library [20]. The QUADAS‐2 tool was used to assess the risk of bias and the applicability of the study to answer the research question in four domains: patient selection, index test, reference standard, flow and timing (see Fig. S1). Each domain was evaluated as low risk, unclear risk, or high risk of bias.

The global ability to evaluate performance of biomarker(s) for CRC screening was determined through a four‐step algorithm. First, design of the study was analyzed. Patient‐control designs were considered as poor‐ability studies. The other studies moved to the second step. Second, the type of included CRC was checked. Studies that included prevalent CRC were considered as poor‐ability studies. The other studies moved to a third step. Third, the type of included controls was checked. Studies that included control patients who did not undergo colonoscopy were considered as poor‐ability studies. The other studies moved to the fourth step. Finally, risk of bias and applicability concerns following the QUADAS score and statistical validation were analyzed. Studies with a majority of light gray rectangles in the QUADAS score, and with a statistical validation method, were considered as good‐ability studies. Studies with a majority of black/dark rectangles in the QUADAS score, or without a statistical validation method, were considered as medium‐ability studies.

### 2.5 Statistical analysis

The estimation of the AUC for CRC or AN screening reported in the included studies for each biomarker or combination of biomarkers was represented through forest plots, with a color code indicating the global ability to evaluate biomarkers for CRC screening (black square for poor ability, gray square for medium ability and white square for good ability). r software (Vienna, Austria) was used to perform the analysis and construct the forest plots.

## 3 Results

### 3.1 Study selection

**Fig. 1:**
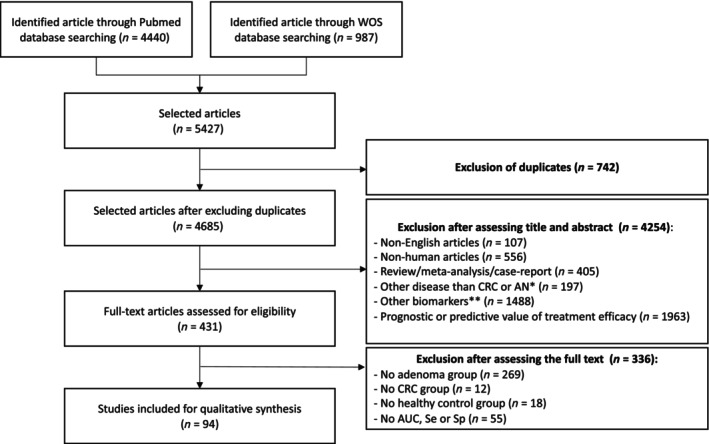
Flow chart of studies included in this systematic review. *Other cancers than CRC, other colorectal diseases (IBD, diverticulitis…), obesity, diabetes. **Stool biomarkers only, urinary biomarkers, circulating DNA/RNA/microRNA biomarkers, circulating cells biomarkers. AN, advanced neoplasia; AUC, area under the curve; CRC, colorectal cancer; Se, sensitivity; Sp, specificity; WOS, Web of Science.

A literature search was conducted on May 6, 2024, and 4685 articles published between January 2010 and December 2023 were identified; 431 of these articles were eligible for inclusion in the review. After a full‐text assessment for inclusion, only 94 studies were included [21, 22, 23, 24, 25, 26, 27, 28, 29, 30, 31, 32, 33, 34, 35, 36, 37, 38, 39, 40, 41, 42, 43, 44, 45, 46, 47, 48, 49, 50, 51, 52, 53, 54, 55, 56, 57, 58, 59, 60, 61, 62, 63, 64, 65, 66, 67, 68, 69, 70, 71, 72, 73, 74, 75, 76, 77, 78, 79, 80, 81, 82, 83, 84, 85, 86, 87, 88, 89, 90, 91, 92, 93, 94, 95, 96, 97, 98, 99, 100, 101, 102, 103, 104, 105, 106, 107, 108, 109, 110, 111, 112, 113, 114] (Fig. 1). A total of 336 studies were excluded: 269 because of the lack of an adenoma or a polyp group and 55 because of the lack of communication of AUC, sensitivity, or specificity for the studied biomarker(s).

### 3.2 Study characteristics

The most common designs were case–control studies (49/94, 52.1%), retrospective analysis of biobanks (21/94, 22.3%), and prospective recruitment for colonoscopy (24/94, 25.5%), including only two studies restricted to FIT‐positive patients [53, 74] (Table 1).

**Table 1:** Characteristics of the included studies.

Study (authors/year)	Design^a^	Statistical validation^b^	Population of the study					Number of biomarkers tested	CEA assessment^g^	QUADAS‐score^h^							Global ability to assess biomarkers for CRC screening^i^
			Sample size (CRC + AA)	Mean age (years)^c^ (CRC/adenoma/HC)	CRC or adenoma history^d^	Type of included CRC^e^	Colonoscopy in controls^f^			Risk of bias				Applicability concerns			
										Patient selection	Index test	Reference standard	Flow and timing	Patient selection	Index test	Reference standard	
Attallah 2018	P	None	235 (150 + NG)	NP	NP	2	0	3	1								Poor
Atwa 2020	P	None	251 (120 + NG)	48 (51/44/49)	0	2	0	1	0								Poor
Bedin 2015	C	None	151 (90 + 27)	65 (68/61/62)	NP	3	1	88	0								Poor
Bhardwaj 2020a	B	T + V (bs)	457 (154 + 101)	65 (66/65/65)	NP	1	1	275	0								Good
Bhardwaj 2020b	B	T + V (bs)	450 (156 + 99)	65 (66/65/65)	NP	1	1	275	0								Good
Bünger 2012	B	T + V (bs)	317 (164 + NG)	66 (70/64/62)	NP	1	1	9	1								Good
Cai 2022	C	T + V	288 (96 + NG)	57 (56/57/57)	NP	3	1	4	1								Poor
Chen H 2016	B	T + V	753 (401 + 99)	65 (66/64/62)	NP	1	1	64	0								Good
Chen H 2017	B	T + V	598 (267 + NG)	65 (68/64/62)	NP	1	1	5	0								Good
Chen M 2020	C	None	601 (301 + NG)	62 (62/62/61)	NP	3	1	4	0								Poor
Christensen 2015	B	None	1965 (32 + NG)	59 (67/65/56)	NP	2	1	2	1								Med
Dai 2021	P	None	90 (19 + 47)	58 (56/55/50)	1	1	1	4	1								Med
De Chiara 2010	P	None	299 (33 + 50)	NP	NP	2	1	1	0								Med
De Chiara 2022	P	None	1703 (249 + 372)	62 (NP)	NP	2	1	2	0								Med
Deng 2013	C	T + V	187 (67 + NG)	62 (60/58/55)	NP	3	1	4	1								Poor
Dowling 2014	C	T + V	106 (56 + NG)	62 (62/60/65)	NP	2	1	4	0								Poor
Dressen 2017	C	None	106 (35 + NG)	52 (69/55/39)	NP	3	NP	24	1								Poor
Farshidfar 2016	B	T + V	605 (320 + NG)	64 (65/60/62)	NP	3	1	48	0								Poor
Fitzgerald 2018	P	None	114 (24 + NG)	67 (67/67/67)	NP	2	1	7	0								Med
Garranzo‐A. 2019	B	None	96 (40 + NG)	59 (66/59/61)	NP	3	1	9	0								Poor
Gawel 2019	B	T + V	4493 (512 + 399)	NP	NP	2	1	8	1								Good
Gimeno‐G. 2015	P	None	150 (25 + 25)	63 (68/63/62)	0	2	1	2	0								Med
Groblewska 2010	C	None	210 (91 + NG)	NP	NP	3	NP	4	1								Poor
Gu J 2019	C	None	110 (40 + NG)	NP	NP	3	0	23	0								Poor
Gu Y 2022	C	None	382 (120 + 130)	60 (62/59/60)	NP	3	0	5	1								Poor
Han 2014	C	None	174 (113 + NG)	61 (62/58/60)	NP	3	NP	2	1								Poor
Huang Z 2022	C	None	460 (227 + NG)	62 (NP)	NP	3	NP	3	1								Poor
Huang M 2023	P	None	291 (101 + NG)	58 (67/55/41)	NP	2	1	3	1								Med
Ivancic 2020	C	T + V (cv)	259 (47 + 72)	60 (NP)	NP	3	1	17	0								Poor
Jiang 2021	C	T + V	1729 (148 + NG)	NP	NP	3	1	2	1								Poor
Johansen 2014	P	None	4496 (293 + NG)	61 (NP)	1	2	1	2	1								Med
Ke 2023	C	T + V	209 (74 + NG)	62 (65/62/58)	NP	3	0	6	1								Poor
Kleif 2022	P	None	4048 (242 + 548)	NP	NP	1	1	9	1								Med
Kraus 2015	C	T + V	297 (98 + NG)	59 (63/62/55)	0	3	1	1	0								Poor
Li Q 2016	C	None	204 (127 + NG)	64 (66/62/60)	0	3	1	2	1								Poor
Li S 2019	C	None	327 (160 + NG)	NP	NP	3	NP	2	1								Poor
Li B 2020	C	T + V	250 (165 + NG)	54 (56/52/54)	0	1	1	1	0								Poor
Liu L 2020	C	None	313 (151 + NG)	60 (61/57/60)	NP	3	1	3	1								Poor
Liu Z 2021	C	T + V	243 (120 + NG)	NP	NP	3	0	4	0								Poor
Meng 2012	P	None	483 (93 + 41)	59 (59/60/57)	NP	3	1	2	1								Poor
Montero‐C. 2023	C	None	110 (31 + NG)	67 (71/64/67)	NP	3	0	2	0								Poor
Moravkova 2019	C	None	84 (22 + 22)	63 (69/64/55)	0	3	1	4	0								Poor
Mroczko 2010	C	None	180 (75 + NG)	NP	NP	3	NP	3	1								Poor
Nielsen 2011	P	None	4486 (294 + NG)	61 (NP)	NP	2	1	1	0								Med
Otero‐E. 2014	P	None	516 (4 + 53)	54 (NP)	1	1	1	1	0								Med
Otero‐E. 2015	P	None	516 (4 + 53)	54 (NP)	1	1	1	1	0								Med
Otero‐E. 2016	P	None	511 (4 + 53)	NP	1	1	1	1	0								Med
Overholt 2015	P	None	448 (134 + NG)	57 (NP)	NP	3	1	1	0								Poor
Ozemir 2016	C	None	80 (27 + 8)	56 (NP)	NP	3	1	3	1								Poor
Ozgur 2019	P	None	185 (40 + NG)	57 (61/59/55)	NP	2	1	2	0								Med
Pan Y 2021	C	T + V	362 (163 + 98)	60 (61/60/58)	NP	3	0	52	1								Poor
Pan Z 2022	C	T + V	127 (65 + NG)	56 (57/55/55)	NP	3	NP	3	1								Poor
Peltier 2016	B	T + V	85 (24 + NG)	61 (67/66/53)	NP	3	1	3	0								Poor
Petersen 2023	P	None	4048 (242 + 548)	65 (68/66/63)	0	1	1	9	1								Med
Qian 2018	B	T + V	651 (45 + 120)	NP	NP	1	1	1	0								Good
Qiu 2019	P	None	164 (20 + NG)	60 (64/67/57)	0	2	1	1	0								Med
Rasmussen 2021	B	None	784 (196 + 98)	66 (70/66/64)	0	1	1	1	0								Med
Rho 2016	B	T + V	1179 (653 + NG)	NP	NP	2	1	5	0								Good
Rigi 2020	C	None	178 (56 + NG)	57 (NP)	1	3	1	1	0								Poor
Solé 2015	C	None	180 (80 + NG)	66 (66/60/67)	NP	3	1	1	0								Poor
Song Y 2016	C	None	382 (137 + NG)	58 (59/57/57)	NP	3	0	3	1								Poor
Song W 2020	C	None	1010 (350 + NG)	NP	NP	3	0	37	1								Poor
Storm 2015	C	T + V	991 (99 + NG)	63 (69/65/62)	0	3	1	3	0								Poor
Sun 2018	C	None	1365 (455 + NG)	57 (58/57/55)	NP	3	0	4	1								Poor
Taguchi 2015	B	None	180 (60 + NG)	60 (63/62/56)	NP	3	1	3	1								Poor
Tao 2012	B	None	597 (179 + 193)	65 (68/65/62)	NP	2	1	3	0								Med
Thomas 2015	B	None	100 (40 + NG)	62 (62/62/62)	0	2	NP	2	1								Poor
Thorsen 2013	B	T + V (cv)	280 (70 + NG)	72 (72/72/72)	1	2	1	74	1								Med
Uchiyama 2017	C	None	175 (56 + NG)	69 (70/70/68)	NP	3	1	334	0								Poor
Uchiyama 2018	C	T + V	304 (140 + NG)	69 (70/70/68)	NP	3	1	5	0								Poor
Van Den B. 2010	P	None	616 (69 + NG)	60 (69/61/58)	NP	1	1	3	0								Med
Wang J 2013	C	None	366 (201 + 80)	54 (55/52/53)	NP	3	0	2	1								Poor
Wang X 2017	C	None	987 (473 + NG)	NP	NP	3	0	2	1								Poor
Wang D 2019	C	T + V	428 (187 + NG)	57 (58/56/56)	NP	3	NP	3	1								Poor
Wang Z 2021	C	None	173 (81 + NG)	53 (54/47/56)	NP	3	0	2	1								Poor
Wang H 2023	C	None	817 (204 + 186)	57 (58/56/57)	NP	3	0	26	1								Poor
Wang Q 2023	C	None	313 (202 + NG)	NP	0	3	1	3	1								Poor
Watany 2018	C	None	120 (49 + NG)	NP	NP	3	NP	3	1								Poor
Weiss 2011	C	None	178 (59 + NG)	56 (68/45/55)	NP	3	0	2	1								Poor
Werner 2016	B	None	1660 (40 + NG)	62 (66/64/62)	NP	1	1	5	1								Med
Wild 2010	B	T + V	710 (301 + 143)	65 (67/67/62)	NP	3	1	6	1								Poor
Wilhelmsen 2017	P	None	4698 (512 + 399)	NP	0	2	1	8	1								Med
Wilson 2012	P	None	748 (3 + 43)	59 (NP)	NP	2	1	1	0								Med
Wu 2015	B	None	93 (49 + NG)	60 (62/59/56)	NP	3	0	2	1								Poor
Xie 2017	C	T + V	870 (346 + NG)	57 (59/57/54)	NP	3	0	2	1								Poor
Xu 2015	C	None	152 (125 + NG)	NP	NP	3	NP	7	0								Poor
Yao 2012	B	None	215 (122 + NG)	NP	NP	3	NP	2	1								Poor
Ye 2014	C	None	199 (120 + NG)	64 (NP)	NP	3	NP	3	1								Poor
Yildrim 2018	P	None	68 (23 + NG)	59 (63/63/49)	NP	2	1	2	1								Med
Zekri 2015	P	None	114 (34 + NG)	42 (41/40/42)	NP	2	1	3	0								Med
Zhang 2015	C	None	249 (138 + NG)	58 (NP)	NP	3	1	2	1								Poor
Zhou 2019	C	None	460 (258 + NG)	NP	1	3	NP	4	1								Poor
Zhu C 2010	C	None	398 (144 + NG)	66 (68/64/66)	NP	3	1	2	1								Poor
Zhu J 2014	C	T + V (cv)	234 (66 + NG)	57 (58/56/57)	NP	3	1	13	0								Poor

Abbreviations: AA, advanced adenoma; CRC, colorectal cancer; HC, Healthy controls; NG, details of the proportion of advanced or high‐risk adenomas were not given for this adenoma group; NP, not precised.

^a^ P = Prospective, C = Case–control, B = Biobank.

^b^ T + V = training + validation set, bs = bootstrap, cv = cross‐validation.

^c^ Mean age in years in the overall population and in the CRC, the adenoma and the HC subgroups.

^d^ 0 = no personal of family history of adenoma or CRC, 1 = possible personal or family history of CRC.

^e^ 1 = Incident CRC diagnosed screening colonoscopy in asymptomatic patients, 2 = Incident CRC diagnosed after colonoscopy in symptomatic patients, 3 = Prevalent case of CRC.

^f^ 1 = total colonoscopy performed in all healthy controls, 0 = total colonoscopy not performed in all healthy controls.

^g^ CEA assessment was rated as 1 if performed and 0 if not.

^h^ QUADAS score items were rated as high risk (black rectangle), unclear risk (dark gray rectangle) or low risk of bias or applicability concerns (light gray rectangle), following the recommendations of the Cochrane Library [20].

^i^ Global ability to evaluate biomarkers for CRC screening was determined through a four‐steps algorithm, as follows: 1st step: design of the study. Patient‐control designs were considered as poor‐ability studies. The other studies moved to step 2. 2nd step: type of included CRC. Studies that included prevalent CRC were considered as poor‐ability studies. The other studies moved to step 3. 3rd step: type of included controls. Studies that included control patients who did not undergo colonoscopy were considered as poor‐ability studies. The other studies moved to step 4. 4th step: risk of bias and applicability concerns following QUADAS score, and statistical validation. Studies with a majority of light gray rectangles in QUADAS score, and with a statistical validation method, were considered as good‐ability studies. Studies with majority of black/dark rectangles in QUADAS score, or without a statistical validation method, were considered as medium‐ability studies.

Quantitative assay results raise the question of cutoff value selection. There was a high heterogeneity between included studies to determine the cutoff value of candidate biomarkers for CRC screening. The best procedure was based on a 2‐step strategy. First, a training set was used to identify the cutoff value associated with the best performance (sensitivity and specificity), or with the best sensitivity for a prespecified specificity (often set at 90%). Then, performance of the biomarker at this cutoff value was tested through a second cohort, in the validation set. A statistical validation with a training set and a validation set was performed in 28 studies only (29.8%), with six using cross‐validation or bootstrap resampling. Six studies (6.4%) determined the cutoff value based on previous data or results described in the literature for the considered biomarker(s). In this case, the rationale behind the choice of the cutoff value was then clearly explained in Section 2. However, the remaining 60 studies have calculated biomarker performance based on the most interesting cutoff value, without any preexisting rationale, and without statistical validation in a second cohort (63.8%).

The median number of patients included in the studies was 302, ranging from 68 to 4698. Despite the inclusion of studies involving groups of patients with adenoma or polyps, only 28 of them (29.8%) specified the AA (or high‐risk polyp) population. This detail is important for assessing the diagnostic capacity of the test for precancerous lesions, since one in four AAs will develop into cancer. The risk of progression from adenoma to cancer is much lower for simple adenomas than for AA [115].

The mean age of the overall population was available in 73 studies (77.6%) and detailed in the three subgroups in 60 studies (63.8%). Among these studies, the mean age was 61 years for the overall population, 63 years for CRCs, 62 years for adenomas, and 60 years for HC.

Personal or family history of polyp or CRC in the study population was not recorded or detailed in most studies (73/94, 77.6%).

Selection of a study population representative of the asymptomatic patients targeted by CRC‐screening is a crucial point. Among the 94 studies, only 16 included screen‐detected incident CRC (17.0%), which was the targeted CRC population. Other studies included incident CRC diagnosed in symptomatic patients (21/94, 22.3%) or prevalent CRC (57/94, 60.1%). Furthermore, only 62 (66.0%) included a colonoscopy‐validated control group, including two with part of flexible sigmoidoscopy [51, 64]. Nineteen studies had a noncolonoscopy‐validated control group (20.1%), and the results were unclear for 13 other studies (13.9%).

Several exploratory studies have tested numerous biomarkers, with 29 studies evaluating five biomarkers or more (30.1%), which raises the problem of risk alpha inflation.

Numerous studies measured CEA (51/94, 54.3%) and/or CA19‐9 (18/94, 19.1%). These two biomarkers have been extensively studied in the context of CRC screening, but their accuracy in CRC screening is limited. A systematic review reported a sensitivity between 40% and 60% for CRC detection and between 5% and 30% for AA detection and a specificity between 85% and 97% for CEA. The sensitivity of CA19‐9 for detecting CRC was between 16% and 52%, and the specificity was between 80% and 97% [116]. The accuracy of these two biomarkers therefore sheds light on the study carried out and may argue for an overestimation of the accuracy of the candidate biomarker in the case of high accuracy for these two biomarkers.

### 3.3 Risk of bias and applicability of studies

Table 1 also presents the quality assessment and level of evidence of the included studies.

Most of the included studies presented a high or an unclear risk of selection bias (66.0%). This bias was explained by the case–control design, or the absence of description of consecutive or random sample for prospective enrolment. Thus, the cases are not representative of the screening population because of the importance of advanced and/or symptomatic CRC. The proteomic profile of these patients could be different from that of asymptomatic patients with early‐onset CRC (or AA) targeted by CRC screening.

Furthermore, most of the studies also presented measurement bias because the cutoff was established without prior data, and no confirmation was made through a two‐step design with training or validation sets.

The timing of blood collection was unclear or not specified in 39 studies (41.5%), which can lead to confusion concerning the quality of the presented results.

Finally, some techniques for biomarker measurement are difficult to reproduce, raising the question of the external validity of the results, especially for spectrometry techniques.

When analyzing the included studies following our four‐steps algorithm (as described above), only eight studies were classified as good ability to evaluate the performance of the biomarker for CRC screening (8.5%). Twenty‐four were classified as medium‐ability (25.5%), and the remaining 62 studies as poor‐ability (66.0%).

### 3.4 Global assessment of serum protein biomarkers for CRC screening

Several serum protein biomarkers have been identified as potentially interesting for CRC screening (see Table S1). However, these results must be interpreted based on the ability of the studies to answer the question.

**Fig. 2:**
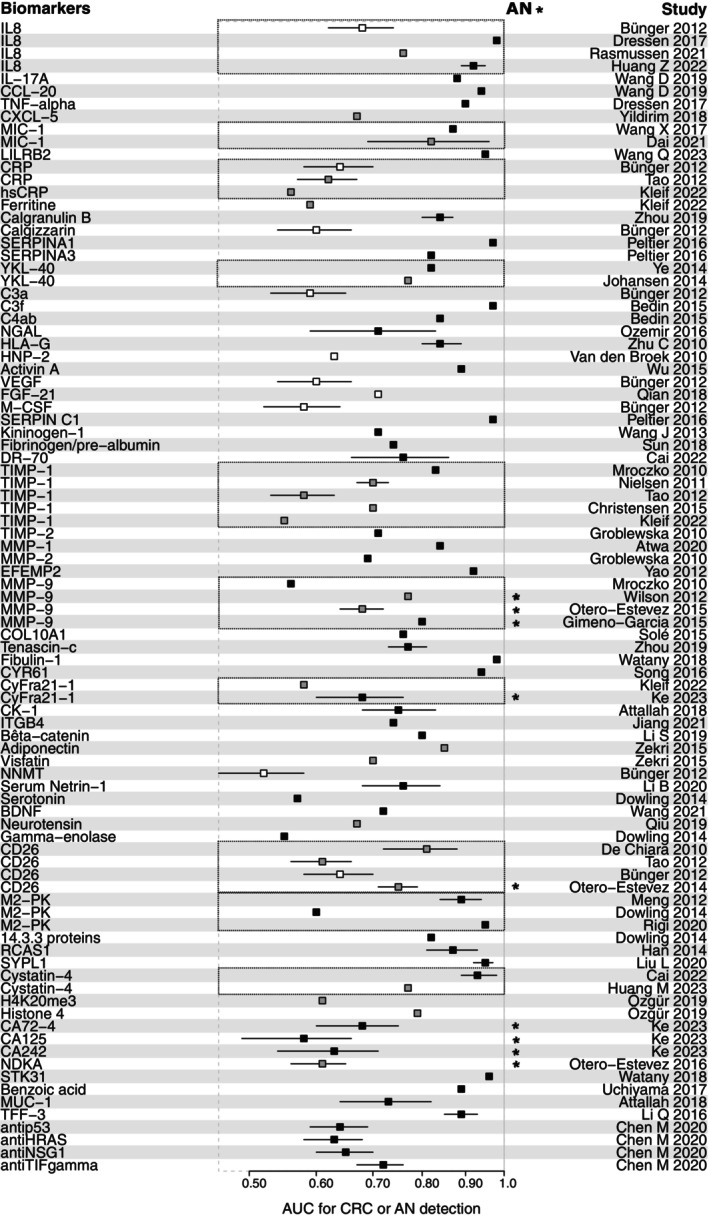
Forest plot representing AUC of the different isolated biomarkers for colorectal cancer or advanced neoplasia detection. *Represents the AUC for advanced neoplasia detection. The white squares represent good‐ability studies, the gray squares represent medium‐ability studies, and the black squares represent poor‐ability studies, as defined by the global ability assessment in Table 1. The AUC was represented by a square when no confidence interval was reported in the study. AN, advanced neoplasia; AUC, area under the curve; CRC, colorectal cancer.

Figure 2 summarizes the AUCs for CRC or AN detection for the main biomarkers identified, with a color code weighing the ability to evaluate biomarkers for CRC screening. This figure highlights the global tendency to overestimate the intrinsic performance of biomarkers, since the majority of results come from studies not properly designed to evaluate biomarkers for CRC screening. All the biomarkers with an AUC greater than 0.90 were identified in case–control or retrospective analyses of biobank studies, with poor ability to evaluate biomarkers for CRC screening (15/15, 100%).

When looking at reported AUCs without weighing for study heterogeneity, the median AUC for CRC or AN detection for tested biomarkers was 0.75, ranging from 0.52 to 0.98. The median AUC was 0.60 for good‐ability studies (ranging from 0.52 to 0.71), 0.69 for medium‐ability studies (ranging from 0.55 to 0.85), and 0.82 for poor‐ability studies (0.55–0.98). The influence of the methodological ability to evaluate biomarkers for CRC screening on the AUC was striking when looking at interleukin (IL)‐8, with an AUC for CRC detection between 0.92 and 0.98 in poor‐ability studies, 0.76 in a medium‐ability study, and only 0.68 in a good‐ability study (Fig. 2). Median AUC was 0.77 in studies without methodological validation (ranging from 0.55 to 0.98) and 0.68 in studies with methodological validation (ranging from 0.52 to 0.97).

When considering results of studies with a good‐ or medium‐ability to evaluate biomarkers for CRC screening, the most promising biomarkers are IL‐8 (AUC = 0.76) [77], MIC‐1 (AUC = 0.82) [32], YKL‐40 (AUC = 0.77) [51], FGF‐21 (AUC = 0.71) [75], TIMP‐1 (AUC = 0.70) [31, 64], MMP‐9 (AUC = 0.77) [103], adiponectin (AUC = 0.85) [110], sCD26 (AUC = 0.81) [33, 65], cystatin 4 (AUC = 0.77) [48] and histone 4 (AUC = 0.79) [70]. However, when comparing these results to the FIT (AUC = 0.95), no single biomarker could currently challenge the fecal test.

### 3.5 Global assessment of combinations of biomarkers for CRC screening

One way of increasing the diagnostic performance of a blood test is to combine several protein biomarkers. Table S2 presents the different combinations of protein biomarkers. Most of these studies were based on the enzyme‐linked immunosorbent assay (ELISA). Several combinations of CEA with one or more other biomarkers of interest were evaluated, with AUCs ranging from 0.75 to 0.99 for CRC detection and from 0.56 to 0.93 for AA detection [31, 32, 50, 51, 52, 57, 58, 64, 72, 81, 84, 85, 95, 96, 100, 101, 105, 108, 111, 112, 113]. Other combinations of 4–11 protein biomarkers measured by different multiplex immunoassays (Proximity Extension Assays [Olink®, Biogenity ApS, Aalbord, Denmark] [24, 78, 88] or the Abbott Architect® i2000R automated platform [41, 53, 102]) have also shown promising results. In addition, serum peptide profiles were studied using various spectrometry techniques, with AUCs ranging from 0.67 to 0.93 for CRC detection and from 0.65 to 0.83 for adenoma detection [25, 35, 38, 44, 45, 49, 72, 90, 106, 114].

**Fig. 3:**
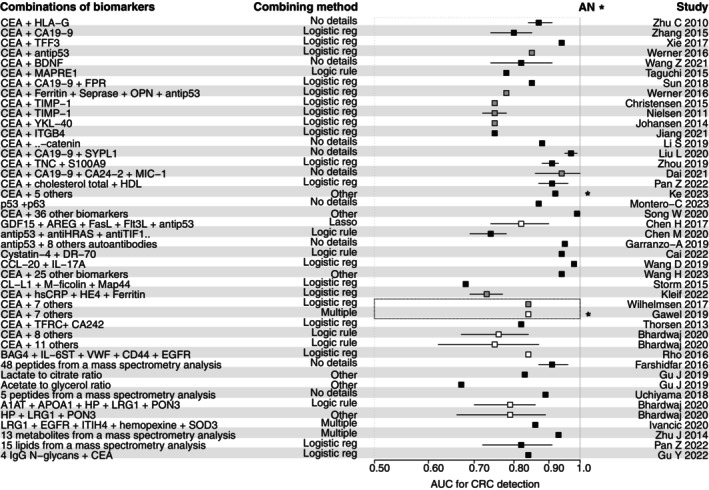
Forest plot representing AUC of the different combination of biomarkers for colorectal cancer or advanced neoplasia detection. *Represents the AUC for advanced neoplasia detection. The white squares represent good‐ability studies, the gray squares represent medium‐ability studies, and the black squares represent poor‐ability studies, as defined by the global ability assessment in Table 1. The AUC was represented by a square when no confidence interval was reported in the study. AN, advanced neoplasia; AUC, area under the curve; CRC, colorectal cancer.

Figure 3 summarizes the AUCs for CRC or AN detection for the main combinations identified, with the color code weighting the ability to evaluate biomarkers for CRC screening. Among the 13 combinations of biomarkers with an excellent diagnostic performance, with an AUC greater than 0.90, only one was identified in a medium‐ability study [32].

When considering the results of good‐ or medium‐ability studies, four combinations of biomarkers presented an AUC between 0.80 and 0.90: CEA + anti‐p53 (AUC = 0.85) [100], CEA + CA19‐9 + AFP + TIMP‐1 + CyFra21‐1 + hsCRP + Ferritin + Galactin‐3 (AUC = 0.84) [41, 102], and BAG‐4 + IL6ST + vWF + CD44 + EGFR (AUC = 0.84) [78]. Only one combination of biomarker presented an AUC higher than 0.90: the association of CEA + CA19‐9 + CA24‐2 + MIC‐1 (AUC = 0.94). This promising result comes from a Chinese study, with an adapted study population [32]. However, no statistical validation method was performed, and confirmation of the performance of this combination in another prospective cohort is pending. Thus, no combination of biomarkers could currently challenge the FIT in the diagnostic ability for CRC screening.

### 3.6 Combination of serum protein biomarkers with the FIT for CRC screening

Another way of increasing the performance of a screening program would be to combine a blood screening test with a fecal test (immunological Fecal Occult Blood Test or FIT) (Table S3). However, this solution has been understudied. One study assessing the sCD26 + FIT combination found an interesting sensitivity of 64.9% for AN detection and 62.3% for AA detection at 90% specificity, with a FIT cutoff of 20 μg Hb·g^−1^ feces [65]. When combining sCD26, DDP4, and FIT results through a decision algorithm, the sensitivity was 95.2% for CRC detection and 80.3% for AA detection. Thus, a sCD26/DDP4 blood test could be proposed after a FIT‐positive test to avoid unnecessary colonoscopy. However, this algorithm must be validated in a prospective study [34]. A recent Danish study found a better AUC for a two‐step algorithm approach combining FIT, patients characteristics (age and sex), and serum biomarkers (CEA, hsCRP, Ferritin, TIMP‐1, Pepsinogen‐2, HE4, Cyfra21‐1, Galectin‐3, and β2‐microglobulin) as compared with FIT alone, with a cutoff value set at 100 ng·mL^−1^ of hemoglobin (AUC = 0.75 [0.72–0.78] *vs* AUC = 0.69 [0.66–0.72], respectively) [74]. These two recent works open up the prospect of a decision‐making algorithm based on the combination of the FIT result and one or a combination of more routinely assayable protein biomarkers of interest, making it possible to increase the performance of the screening program.

## 4 Discussion

This systematic review reports the most interesting studies assessing serum protein biomarkers for CRC, AN, or AA detection in the past 13 years. A recent consensus of international experts reported that an accuracy as good as that of the FIT is needed to determine whether blood protein biomarkers are an option [117]. Thus, these biomarkers should have at least a 0.95 AUC for CRC detection, which corresponds to 79% sensitivity and 94% specificity [5]. Despite the extensive literature in this field, there are currently no serum protein biomarkers nor combinations of biomarkers that present sufficient accuracy for replacing the FIT.

### 4.1 How to conduct new studies assessing biomarkers for CRC screening?

This review highlights the significant heterogeneity of the study population, despite stringent inclusion criteria. Most of the included studies were case–control studies, with prevalent CRC, which overestimate the performance of candidate biomarkers. The question of selecting a study population representative of the population targeted by screening is crucial. The performance of a protein biomarker for CRC screening can only be properly assessed in a population of asymptomatic patients targeted by CRC screening [18]. This review highlights the importance of conducting better‐designed studies. Some European biological collections, such as the German BLITZ cohort [24] or the Danish cohort [53], are currently the best studies to rely on in terms of methodological examples to assess the usefulness of biomarkers for CRC or AN detection. These cohorts included many asymptomatic patients referred for a screening colonoscopy.

In our opinion, future studies conducted on the subject should follow the recent guidelines for the assessment of new noninvasive screening tests for CRC [117]. Prospective studies are to be preferred to case–control studies, with a low level of evidence and a high risk of bias. When performing a prospective study, only asymptomatic patients referred for a screening colonoscopy must be included. If a case–control study, only incident CRC diagnosed in asymptomatic patients after a screening colonoscopy, must be included. Patients in the control group must have undergone a total colonoscopy and be eligible for CRC screening. Then, timing of the blood sampling and the preanalysis process must be clearly defined to be able to reproduce the study.

Furthermore, our review underlines the heterogeneity of included studies to determine the cutoff value. Only 29.8% of the included studies have the recommended two‐step strategy, with the establishment of the cutoff value in a first training cohort, and the statistical validation of this value in a second cohort. This two‐step design must be encouraged in future studies, to be able to compare the results. Data sharing, with detailed biomarker results and study population characteristics, should be encouraged. Indeed, 50% of the studies included had fewer than 302 patients, which raises the problem of a lack of power, potentially explaining the disappointing results. The sharing of detailed, open‐access data would enable an individual participant data network meta‐analyses to be carried out, taking into account the heterogeneity of the study population due to the characteristics transmitted, and thus coupling the efforts of different teams to make progress in this field of research.

Another issue is the presence of publication bias in the field of serum protein biomarkers for CRC. The tendency to publish significant or favorable results while neglecting null findings can distort the overall understanding of biomarker performance and hinder reproducibility. Efforts to mitigate publication bias, such as establishment of registries and journal that accept null findings, has to be encouraged [118].

### 4.2 Heterogeneity of the methodological process used to combine biomarkers: which statistical method is the best?

Calculating the diagnostic performance of biomarker combinations is also a fundamental issue. Several statistical methods could be used. The most frequently used tool was the construction of an ROC curve using a multivariate logistic regression model that included biomarkers. A least absolute shrinkage and selection operator (LASSO) logistic regression model was used in some studies. Another intuitive method, “and‐or combination,” following logic combination rules could be used [119]. A combination was also considered positive if one or more of the biomarkers were positive or if each biomarker was positive. However, these techniques are not suitable for combinations of numerous biomarkers. More complex analysis models, such as Monte Carlo cross‐validation, partial least‐squares discriminant analysis, or support vector machine analysis, are then needed.

### 4.3 Future serum protein biomarkers: which way to look?

Among the serum protein biomarkers, different families of serum proteins are interesting because of their proven or suspected involvement in colorectal tumorigenesis (Table S1). This systematic review updates known data on serum protein biomarkers for CRC screening, one of the currently explored pathways for the development of a blood‐based CRC screening test. The conclusions are similar to those of the review conducted by Liu *et al*. [17] a decade ago; namely, that there is currently no credible serum protein biomarker or combination of serum protein biomarkers to replace FIT. Considering the results of the best‐designed studies, only a combination of biomarkers (associating CEA, CA19‐9 CA24‐2, and MIC‐1) approaches the AUC of FIT with an AUC of 0.94. However, this result comes from a study with a small sample size (90 patients, including 19 CRCs), and needs to be confirmed on a larger cohort of asymptomatic subjects eligible for screening [32].

Despite the so‐far disappointing results, there are still many avenues to explore. First, thanks to new bioassay methods, further studies will be carried out to precisely analyze the proteome and the metabolome of asymptomatic patients to identify a profile associated with the presence of CRC or AA. Then the advent of artificial intelligence is enabling the development of increasingly complex algorithms, which can take into account the results of several serum protein assays, as well as individual patient characteristics.

One of the most promising short‐term avenues seems to be the development of multistage algorithms combining the FIT result with certain protein assays to increase the performance of the screening program. A recent Danish study found that a multistage algorithm combining FIT, clinical features, and routine serum protein assays outperformed FIT alone [74]. However, replacing or adding a blood‐based test in the CRC screening program in countries where organized screening based on a fecal test has already been in place for many years raises serious questions. Blood screening is likely to come up against new barriers to participation, such as the need to go to a healthcare professional to have a sample taken, and some patients' fear of taking a blood sample. Medico‐economic studies will also have to determine the cost‐effectiveness of a blood screening test, which will probably have a difficulty in challenging the low cost of fecal testing. For example, the Epi proColon® test, based on the detection of *SEPT9* gene methylation, the only blood test currently accredited by the US Food and Drug Administration (FDA), is currently far more expensive than the FIT, limiting possible extension to the entire population targeted by screening [15].

### 4.4 Strengths of our review

To our knowledge, this systematic review is the first to focus on the use of serum protein biomarkers for CRC screening by involving studies including CRC patients, adenoma patients, and control subjects. In fact, a biomarker for CRC screening is relevant only if it reveals a preneoplastic lesion or early‐stage CRC and not an advanced or metastatic CRC in a symptomatic patient. This systematic review was conducted following the PRISMA‐DTA guidelines, and the Cochrane QUADAS‐2 tool was used to analyze bias in the included studies.

### 4.5 Limitations of our review

Only studies indexed in the NCBI and Web of Science databases were considered. Studies conducted before January 1, 2010, were not included, as the results from previous studies on serum protein biomarkers for CRC diagnosis conducted before were already reported by another systematic review [17]. However, most of the innovative proteomics and metabolomics techniques were not available at that time. Despite these two limitations, more than 4600 studies were initially screened for inclusion.

This review excluded several articles without a polyp or adenoma group, which may exclude biomarkers of interest for CRC screening. This clearcut choice is based on remarks provided by previous systematic reviews on the subject, which highlight the absence of consideration of a polyp or adenoma group in addition to a healthy control group as a potential bias when analyzing the specificity of a biomarker for the diagnosis of CRC [14, 17]. Indeed, the presence of a control group that does not distinguish healthy controls from polyps or adenomas may induce a bias, limiting polyp specificity. As the prevalence of polyps and adenomas in the general population is between 20% and 30%, this subgroup cannot be neglected [120]. In addition, the presence of an adenoma or advanced adenoma group makes it possible to calculate the sensitivity of the biomarker for the detection of preneoplastic lesions, which is a necessity, given FIT's limited capacity. Finally, the *a priori* distinction of an adenoma group when drafting the study protocol often reflects a study population that is more representative of the population targeted by CRC screening. By excluding studies that do not include these three groups, we focused on studies that are the most likely to answer the question.

This systematic review excluded studies assessing blood protein biomarkers for other cancers, as studies including a group of patients with noncolorectal cancer, patients with colonic polyps, and patients with CRC were considered improbable. However, while a blood protein biomarker has good sensitivity for diagnosing CRC or AA, it is also important to measure its specificity in a wider population, which may have other early‐stage cancers. Indeed, the identification of a promising biomarker or combination of biomarkers should be evaluated in a group of patients with noncolorectal cancer to assess the specificity of these biomarkers. Similarly, a group of patients with other noncancerous colonic diseases (e.g. inflammatory bowel disease [IBD], diverticulitis) should also be included to assess biomarker specificity. The inclusion of patients from these two groups in addition to HCs is therefore a quality criterion and should be encouraged in future studies.

## 5 Conclusion

No serum protein biomarkers—or their combination—is currently able to replace the 0.95 AUC, 65% sensitivity, and 95% specificity of the FIT in CRC screening programs. However, the development of new proteomic or metabolomic techniques would allow us to identify profiles associated with CRC or AN. Most of the studies conducted in these fields had poor designs and several biases. Further studies should focus on asymptomatic individuals targeted by screening to avoid overestimation of sensitivity. Research must continue with better‐designed studies to develop new screening tests based on one or more serum protein biomarkers. Development of new algorithms, based on the FIT results, patients' characteristics, and results of serum protein biomarkers, represent a promising option.

## Conflict of interest

The authors declare no conflict of interest.

## Author contributions

AGr, HG, LG, and FDF designed the study research; AGr performed data extraction and screened all titles and abstracts; AGr and SC reviewed independently the eligibility of articles, collected data from included articles, and assessed the methodological quality of each included article; AGr and AGi performed statistical analyses and figures conception; and AGr, LG, FDF, and HG wrote the article. All authors reviewed the article and approved the final version.

## Supporting information


**Fig. S1.** QUADAS‐2 score used to analyze the methodology of each included study.
**Table S1.** Serum protein biomarkers for detecting colorectal cancer or adenoma.
**Table S2.** Combination of serum protein biomarkers for detecting colorectal cancer or adenoma.
**Table S3.** Combination of serum protein biomarker to fecal immunochemical test for detecting colorectal cancer or adenoma.

## Data Availability

The data presented in the current study are available upon reasonable request from the corresponding author.
