# Supplementary material for: Where do we stand with screening for colorectal cancer and advanced adenoma based on serum protein biomarkers? A systematic review
Source: Mol Oncol. 2024 Sep 30;18(11):2629–48. doi: 10.1002/1878-0261.13734 (PMC11547240; doi:10.1002/1878-0261.13734)
Supplement: Supplementary file 1 — Fig. S1. QUADAS‐2 score used to analyze the methodology of each included study. Table S1. Serum protein biomarkers for detecting colorectal cancer or adenoma. Table S2. Combination of serum protein biomarkers for detecting colorectal cancer or adenoma. Table S3. Combination of serum protein biomarker to fecal immunochemical test for detecting colorectal cancer or adenoma. [file MOL2-18-2629-s001.docx]

**Supplementary Information:**

This supplementary information file contained the QUADAS-2 tool used to evaluate included studies (Figure S1), and three tables detailing the different data available for each biomarker (Table S1), combination of biomarkers (Table S2) and combination of a biomarker with a non-invasive faecal test (Table S3) included in our systematic review.

**Figure S1: QUADAS-2 score used to analyze the methodology of each included study**

**
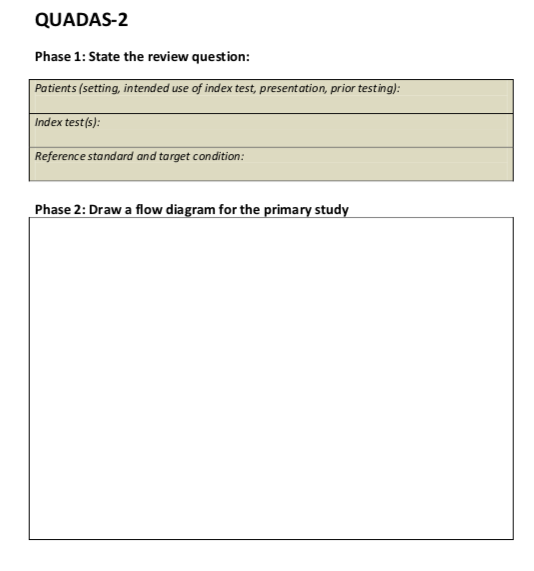
**

**
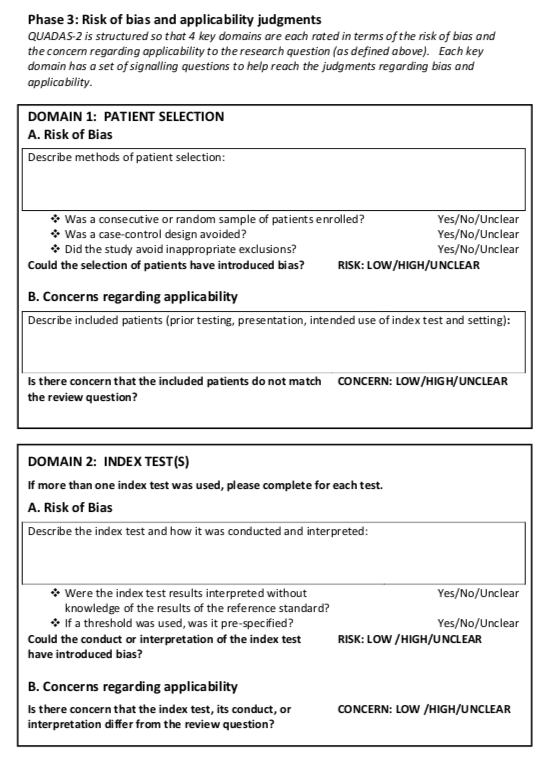
**

**
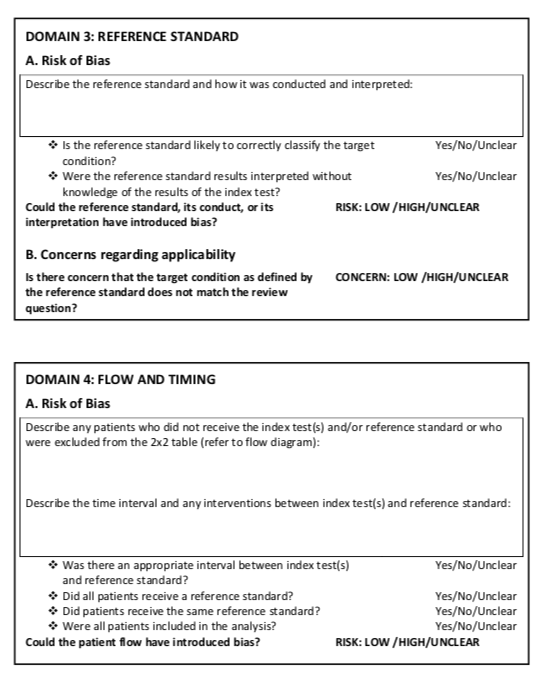
**

**Table S1: Serum protein biomarkers for detecting colorectal cancer or adenoma**

| **Biomarkers** | **Suspected role in tumori-genesis or previous data available** | **Study**  **(year)**  **(country)** | **Design** | **Sample size** | **Statistical validation** | **Technique of measure** | **CRC detection (vs control or adenoma)** | **Comparison early vs late CRC** | **Adenoma detection**  **(vs control)** | **Comparison to other biomarkers or fecal test**  **(AUC for CRC detection)** |
| --- | --- | --- | --- | --- | --- | --- | --- | --- | --- | --- |
| Interleukin 8  (IL-8)  (also known as CXCL-8) | Yes | Bünger (2012)  (Germany) | Retrospective random selection from a German biological collection | 164 CRC, 34 adenomas (12 NAA and 22 AA), 119 HC | Training set and validation set | Development of a nine-biomarkers biochip array divided in two multiplex biochips (CRCSI et II) | ***CRC vs controls:***  AUC = 0.68 (0.62-0.74)  Cut off: 39.5pg/mL  Se = 22%  Sp = 90% | NA | NA | CEA  (AUC=0.69) |
|  | NA | Dressen (2017)  (Germany) | Case-control study | 35 CRC, 20 benign colorectal lesions, 51 HC | No training or validation set | A multiplex immunoassay panel (MILLIPEX Human Circulating Cancer Biomarkers Magnetic Bead Panel) | ***CRC vs controls:***  AUC = 0.98  Se = 85.7%  Sp = 95% | NA | NA | CEA  (AUC=0.92)  CA19-9 (AUC=0.84) |
|  | Yes | Rasmussen  (2021)  (Denmark) | Case-control study with samples from a Danish biological collection | 196 CRC, 98 AA, 98 NAA, 196 non-malignant findings, 196 HC | Discovery study | Olink AB (Stockholm,Sweden) with proximity extension assays for profiling proteins, and quantification by q-PCR. | ***CRC vs others:***  AUC = 0.76 | ***Early-stage CRC vs others (except late-stage CRC)****:*  AUC = 0.73  Se = 58.0%  Sp = 70.0% | NA | NA |
|  | Yes | Huang (2022)  (China) | Retrospective analysis of a Chinese biological collection | 227 CRC, 110 adenomas, 123 HC | No training or validation set | ELISA  (R&D Systems, USA) | ***CRC vs controls:***  AUC = 0.92 (0.89-0.95)  Cut-off: 24.9 pg/mL Se = 86.3%  Sp = 81.3% | NA | NA | CEA  (AUC=0.76) CA19-9 (AUC=0.69) |
| Interleukin 17A (IL-17A) | Yes | Wang  (2019)  (China) | Case-control study of Chinese patients | 112 CRC, 59 HC, 52 Colitis, 40 Adenoma | A screening discovery set, then a training and a validation set | ELISA  (R&D Systems, Minneapolis, MN, USA) | ***CRC vs controls:***  AUC = 0.88  Cut-off : 4.90  Se = 24.8%  Sp = 98.3% | NA | NA | CEA  (AUC=0.60) |
| Chemokine CC ligand 20 (CCL20) also known as Macrophage Inflammatory Protein 3 (MIP3A) | No | Wang  (2019)  (China) | Case-control study of Chinese patients | 112 CRC, 59 HC, 52 Colitis, 40 Adenoma | A screening discovery set, then a training and a validation set | ELISA  (R&D Systems, Minneapolis, MN, USA) | ***CRC vs controls:***  AUC = 0.94  Cut-off : 89.64  Se = 93.1%  Sp = 86.3% | NA | NA | CEA  (AUC=0.60) |
| Tumor Necrosis Factor alpha  (TNF alpha) | NA | Dressen (2017)  (Germany) | Case-control study | 35 CRC, 20 benign colorectal lesions, 51 HC | No training or validation set | A multiplex immunoassay panel (MILLIPEX Human Circulating Cancer Biomarkers Magnetic Bead Panel) | ***CRC vs controls:***  AUC = 0.90  Se = 80%  Sp = 95% | NA | NA | CEA  (AUC=0.92)  CA19-9 (AUC=0.84) |
| Epithelial  neutrophil-activating peptide78 (ENA-78), also known  as CXCL5 | Yes | Yildirim  (2018)  (Turkey) | Prospective recruitment of Turkish patients referred for colonoscopy. | 23 CRC, 22 colonic adenomas, 23 HC. | No training or validation set | ELISA (Bioassay Technology, China) | **CRC vs adenomas:**  AUC = 0.67  Cut-off = 49.6  Se = 57.0%  Sp = 67.0% | NA | NA | CEA  (AUC= 0.69) |
| Macrophage-inhibitory-cytokine-1 (MIC-1) | Yes | Wang  (2017)  (China) | Case-control study of Chinese patients | 473 CRC, 25 benign adenomas, 489 HC | No training or validation set | House sandwich ELISA, which was  produced by CICAMS. | ***CRC vs HC****:*  AUC = 0.87  Cut-off: 1000 pg/mL  Se = 43.8%  Sp = 96.7% | NA | NA | CEA  (AUC=0.73) |
|  | Yes | Dai (2021)  (China) | Prospective recruitment of asymptomatic Chinese patients referred for a screening colonoscopy | 19 CRC, 47 precancerous lesions, 24 randomly selected HC | No training or validation set | ELISA | ***CRC vs controls:***  AUC = 0.82 (0.69-0.96)  Cut-off: 357.64 pg/mL  Se = 82.4%  Sp = 70.8% | NA | ***Precancerous lesions vs controls:***  AUC = 0.76 (0.64-0.88)  Cut-off: 314.1 pg/mL  Se = 54.2%  Sp = 91.5% | CEA  (AUC=0.74)  CA19-9 (AUC=0.66) |
| Leukocyte immunoglobulin-like receptor  B2 (LILRB2) protein | Yes | Wang  (2023)  (China) | Case-control study of Chinese patients | 202 CRC, 93 adenomas, 18 HC | No training or validation set. | Human LILRB2 ELISA Kit (Abcam, Cat No. ab269551). | ***CRC vs controls****:*  AUC= 0.95  Se= 89.7%  Sp = 88.9% | ***NA*** | ***NA*** | CEA  (AUC= 0.75)  CA19-9  (AUC=0.70) |
| C-reactive protein (CRP) | Yes | Bünger (2012)  (Germany) | Retrospective random selection from a German biological collection | 164 CRC, 34 adenomas (12 NAA and 22 AA), 119 HC | Training set and validation set | Development of a nine-biomarkers biochip array divided in two multiplex biochips (CRCSI et II) | ***CRC vs controls:***  AUC = 0.64 (0.58-0.70)  Cut off: 14,600 ng/mL  Se = 17% Sp = 90% | NA | NA | CEA  (AUC=0.69) |
|  | Yes | Tao  (2012)  (Germany) | Retrospective random recruitment from two German biological collections | 179 CRC, 193 AA, 225 HC | No training or validation set | ELISA, Bender Medsystems (Vienna, Austria) | ***CRC vs controls:***  AUC = 0.62 (0.57-0.67)  Cut-off: 2499.9 ng/mL  Se = 19.4%  Sp = 90.3% | NA | ***AA vs controls:***  AUC = 0.50 (0.45-0.55)  Cut-off: 2499.9 ng/mL  Se = 4.9%  Sp = 90.3% | **FIT**  AUC = 0.90  Cut-off: 24 ng/mL  Se = 65.7%  Sp = 97.7% |
| hsCRP | Yes | Kleif  (2022)  (Denmark) | Prospective recruitment of asymptomatic Danish patients with a positive FIT | 242 CRC, 2064 adenomas, 1742 HC | Training set (validation set not published yet) | ELISA  (Abbott Laboratories, USA) | ***CRC vs controls:***  AUC = 0.56 | NA | NA | CEA  (AUC=0.60) |
| Ferritin | Yes | Kleif  (2022)  (Denmark) | Prospective recruitment of asymptomatic Danish patients with a positive FIT | 242 CRC, 2064 adenomas, 1742 HC | Training set (validation set not published yet) | ELISA  (Abbott Laboratories, USA) | ***CRC vs controls:***  AUC = 0.59 | NA | NA | CEA  (AUC=0.60) |
| Calgranulin A  (Protein S100A8)  (a component of calprotectin) | Yes | Moravkova (2019)  (Czech Republic) | Case-control study | 22 CRC, 22 AA, 20 NAA, 20 HC | No training or validation set | ELISA  (Wuhan USCN Life, China) | ***CRC vs controls:***  Se = 94.0%  Sp = 73.0% | NA | NA | NA |
| Calgranulin B  (Protein S100A9) | Yes | Zhou  (2019)  (China) | Case-control study of Chinese patients | 258 CRC, 99 benign lesion (IBD and polyps), 103 HC | No training or validation set | ELISA  (Cusabio, China) | ***CRC vs HC:***  AUC = 0.84 (0.80-0.87)  Cut-off: 18.22 ng/mL  Se = 65.9%  Sp = 89.1% | NA | NA | CEA  (AUC=0.74)  CA19-9  (AUC=0.66) |
| Calgizzarin (S100A11) | NA | Bünger (2012)  (Germany) | Retrospective random selection from a German biological collection | 164 CRC, 34 adenomas (12 NAA and 22 AA), 119 HC | Training set and validation set | Development of a nine-biomarkers biochip array divided in two multiplex biochips (CRCSI et II) | ***CRC vs controls:***  AUC = 0.60 (0.54-0.66) | NA | NA | CEA  (AUC=0.69) |
| SERPIN A1  (also known as alpha-1-antitrypsine) | Yes | Peltier  (2016)  (France) | Retrospective random selection from a French biological collection | Proteomic analysis (Discovery set):  15 CRC, 15 adenomas, 15 HC.  Validation set:  19 CRC, 21 HC | Discovery set and validation set | Discovery phase: iTRAQ  + Reversed-phase liquid chromatography and MALDI TOF/TOF analysis + LTQ Orbitrap.  Validation set: ELISA (PID# KA0458, KA2128 and KA1836,  Abnova) | ***CRC vs HC****:*  AUC = 0.97  Cut-off: 817 μg/mL  Se = 95.0%  Sp = 95.0% | NA | NA | NA |
| SERPIN A3  (also known as alpha-1-antichymotrypsine) | Yes | Peltier  (2016)  (France) | Retrospective random selection from a French biological collection. | Proteomic analysis (Discovery set):  15 CRC, 15 adenomas, 15 HC  Validation set:  19 CRC, 21 HC | Discovery set and validation set | Discovery phase: iTRAQ  + Reversed-phase liquid chromatography and MALDI TOF/TOF analysis + LTQ Orbitrap.  Validation set: ELISA (PID# KA0458, KA2128 and KA1836,  Abnova) | **CRC vs HC**:  AUC = 0.82  Cut-off: 235 μg/mL  Se = 95.0%  Sp = 55.0% | NA | NA | NA |
| YKL-40 (or Chitinase-3-like 1 CHI3L1) | Yes | Ye  (2014)  (China) | Case-control study of Chinese patients | 120 CRC, 43 benign colorectal lesions, 36 HC | No discovery or validation set | ELISA  (Quidel Corporation, USA) | ***CRC vs controls:***  AUC = 0.82  Cut-off: 72 ng/mL  Se = 73.2%  Sp = 66.7% | NA | NA | CEA  (AUC=0.85)  CA19-9  (AUC=0.79) |
|  | Yes | Johansen (2015)  (Denmark) | Prospective recruitment of symptomatic or asymptomatic Danish patients referred for a colonoscopy | 293 CRC, 854 adenomas, 1176 other non-malignant diseases, 2173 HC | No training or validation set | ELISA (Quidel, USA) | ***CRC vs HC:***  AUC = 0.77 | NA | ***Adenomas vs HC:***  AUC = 0.67 | CEA  (AUC=0.76) |
| Complement component 3a (C3a) | NA | Bünger (2012)  (Germany) | Retrospective random selection from a German biological collection | 164 CRC, 34 adenomas (12 NAA and 22 AA), 119 HC | Training set and validation set | Development of a nine-biomarkers biochip array divided in two multiplex biochips (CRCSI et II) | ***CRC vs controls:***  AUC = 0.59 (0.53-0.65) | NA | NA | CEA  (AUC=0.69) |
| Complement component C3f  (peptid 2021.10) | NA | Bedin (2015)  (Italy) | Case-control study | 67 CRC, 27 adenomas, 23 metastatic CRC, 34 HC | No training set, no validation set | Petidome analysis using Nanoporus Silica Chips and MALDI-TOF peptid-profiling | ***CRC vs controls:***  AUC = 0.97 | NA | NA | NA |
| Complement component C4ab  (peptid 1896.03) | NA | Bedin (2015)  (Italy) | Case-control study | 67 CRC, 27 adenomas, 23 metastatic CRC, 34 HC | No training set, no validation set | Petidome analysis using Nanoporus Silica Chips and MALDI-TOF peptid-profiling | ***CRC vs controls:***  AUC = 0.84 | NA | NA | NA |
| Serum Neutrophil Gelatinase Associated Lipocalin (NGAL)  (also known as Lipocalin-2) | Yes | Ozemir  (2016)  (Turkey) | Case-control study | 27 CRC, 24 adenomas, 29 HC | No training or validation set | ELISA  (Santa Clara, CA Cat. NoSK00233-01) | ***CRC vs HC:***  AUC = 0.71 (0.59-0.83)  Cut-off: 104 ng/mL  Se = 71.4%  Sp = 68.9% | NA | NA | CEA CA19-9 |
| Soluble Human Leukocyte Antigen-G (sHLA-G) | Yes. | Zhu  (2010)  (China) | Prospective recruitment of patients referred for CRC surgery or colonoscopy. | 144 CRC, 65 adenomas, 72 HP, 57 IBD, 60 HC | No training or validation set | ELISA (Exbio, Prague, Czech Republic) | **CRC vs controls (hyperplastic polyp, IBD and adenoma):**  AUC = 0.84 (0.80-0.89)  Cut-off: 89 U/mL  Se = 72.2%  Sp = 87.8% | NA | NA | CEA  (AUC=0.65) |
| Human Neutrophil peptide-2 (HNP-2) | Yes | Van den Broek  (2010)  (Netherlands) | Prospective recruitment of Dutch patients referred for colonoscopy in two centers | 69 CRC, 179 adenomas, 368 controls (HC + HP) | No training or validation set | Liquid-chromatographic  method with tandem-mass  spectrometric detection (LCMS/  MS) | ***CRC vs controls****:*  AUC = 0.63  Cut-off: 208.4 ng/mL  Se = 63.8%  Sp = 61.4% | NA | NA | NA |
| Activin A (belonging to the TGF- β superfamiliy) | Yes | Wu  (2015)  (China) | Case-control study from a Chinese biological collection | 49 CRC, 24 benign colorectal polyps, 20 HC | No training or validation set | ELISA  (R&D Systems, Minneapolis, MN,  USA) | ***CRC vs HC****:*  AUC = 0.89  Se = 75.5%  Sp = 85.0% | NA | NA | CEA  (no AUC  Se = 80.0%,  Sp = 63.0%) |
| Vascular endothelial growth factor (VEGF) | Yes | Bünger (2012)  (Germany) | Retrospective random selection from a German biological collection | 164 CRC, 34 adenomas (12 NAA and 22 AA), 119 HC | Training set and validation set | Development of a nine-biomarkers biochip array divided in two multiplex biochips (CRCSI et II) | ***CRC vs controls:***  AUC = 0.60 (0.54-0.66) | NA | NA | CEA  (AUC=0.69) |
| Fibroblast Growth Factor 21 (FGF 21) | Yes  (in other localizations) | Qian  (2018)  (Germany) | Case-control study of German patients. | 45 CRC, 80 AA, 72 NAA, 250 HC. | Discovery set and validation set. | Proseek Multiplex  Inflammatory I Kit (Olink Bioscience, PEA ) and quantified by the  Fluidigm BioMark HD real-time PCR platform | ***CRC vs HC****:*  AUC = 0.71  Se = 45.7 %  Sp = 80.0 %  ***AN vs HC****:*  AUC = 0.61  Se = 24.8%  Sp = 80.0% | NA | NA | NA |
| Macrophage colony-stimulating factor (M-CSF) | Yes | Bünger (2012)  (Germany) | Retrospective random selection from a German biological collection | 164 CRC, 34 adenomas (12 NAA and 22 AA), 119 HC | Training set and validation set | Development of a nine-biomarkers biochip array divided in two multiplex biochips (CRCSI et II) | ***CRC vs controls:***  AUC = 0.58 (0.52-0.64) | NA | NA | CEA  (AUC=0.69) |
| SERPIN C1 (also known as Antithrombin III) | Yes | Peltier  (2016)  (France) | Retrospective random selection from a French biological collection | Proteomic analysis (Discovery set):  15 CRC, 15 adenomas, 15 HC.  Validation set:  19 CRC, 21 HC | Discovery set and validation set | Discovery phase: iTRAQ  + Reversed-phase liquid chromatography and MALDI TOF/TOF analysis + LTQ Orbitrap.  Validation set: ELISA (PID# KA0458, KA2128 and KA1836,  Abnova) | **CRC vs HC**:  AUC = 0.97  Cut-off: 339 µg/mL  Se = 95.0%  Sp =95.0% | NA | NA | NA |
| Kininogen-1 (KNG-1) | Yes | Wang  (2013)  (China) | Case-control study of Chinese patients | 143 pre-operative CRC, 58 post-operative CRC, 80 AA, 85 HC | Training and validation set for the proteomic analysis only | ELISA  (No. EK2001-1, Assaypro, MO, USA) | ***CRC vs HC****:*  AUC = 0.71  Cut-off: 173.96 µg/mL  Se = 63.6%  Sp = 65.9% | ***Early stage (Duke A/B) vs controls:***  Se = 70.1%  Sp = 65.9%  ***Late stage (Duke C/D) vs controls****:*  Se = 72.7%  Sp = 65.9% | ***AA vs HC****:*  AUC = 0.64  Cut-off = 162.99 µg/mL  Se = 51.3%  Sp = 63.5% | CEA  (AUC=0.70) |
| Fibrinogen to pre-albumin Ratio (FPR) | No | Sun  (2018)  (China) | Case-control study of Chinese patients | 455 CRC, 455 benign colorectal polyps, 455 HC | No training or validation set. | SYSMEX CA-7000  Machine (Fibrinogen) + OLYMPUS AU5400 (Pre-Albumin) | **CRC vs HC:**  AUC = 0,74  Cut-off: 15,36  Se = 54.1%  Sp = 81.3%  **CRC vs benign disease:**  AUC = 0,80  Cut-off: 12.25  Se = 72.7%  Sp = 74.3% | NA | NA | CEA  (AUC=0.75)  CA19-9 (AUC=0.65) |
| DR-70 (a fibrin degradation product) | Yes | Cai  (2022)  (China) | Case-control study from a Chinese retrospective cohort | 96 early CRC, 96 benign colorectal lesion, 96 HC | Training set and validation set | ELISA  (Jiangsu Weizhen Bio-pharmaceutical Technology Co**.,** Ltd, China, CFDA 20193400068) | ***CRC vs controls:***  AUC = 0.76 (0.66-0.86)  Se = 28.1%  Sp = 92.2% | NA | NA | CEA  CA19-9 |
| Tissue inhibitor of Metalloproteinase 1 (TIMP-1) | Yes | Mroczko  (2010)  (Poland) | Case-control study | 75 CRC, 35 adenomas, 70 HC | No training or validation set | ELISA  (R&D Systems, England) | ***CRC vs HC:***  AUC = 0.83 | NA | NA | CEA  (AUC=0.83)  CA19-9 (AUC=0.56) |
|  | Yes | Nielsen  (2011)  (Denmark) | Prospective recruitment of symptomatic Danish patient referred for colonoscopy | 294 CRC, 843 adenomas, 1176 non neoplasic findings, 2173 HC | No training or validation set | ELISA  (Architect i2000, Abbott Laboratories, Germany) | ***CRC vs controls:***  AUC = 0.70 (0.67-0.73) | NA | NA | CEA  (AUC=0.73) |
|  | No | Tao  (2012)  (Germany) | Retrospective random recruitment from two German biological collections | 179 CRC, 193 AA, 225 HC | No training or validation set | ELISA, Bender Medsystems (Vienna, Austria) | ***CRC vs controls:***  AUC = 0.58  (0.53-0.63)  Cut-off: 928,2 ng/mL  Se = 13.4%  Sp = 97.7% | NA | **AA vs controls:**  AUC = 0.58 (0.53-0.63)  Se = 7.1%  Sp = 97.7% | **FIT**  AUC= 0.90  Cut-off: 24 ng/mL  Se = 65.7%  Sp = 97.7% |
|  | Yes | Christensen (2015)  (Denmark) | Australian biological collection composed of symptomatic or asymptomatic patients referred for colonoscopy | 32 CRC, 265 adenomas, 1668 controls | Validation study (to confirm prior results) | ELISA (Abbott, Germany) | ***CRC vs controls:***  AUC = 0.70 | NA | NA | CEA  (AUC=0.73) |
|  | Yes | Kleif  (2022)  (Denmark) | Prospective recruitment of asymptomatic Danish patients with a positive FIT | 242 CRC, 2064 adenomas, 1742 HC | Training set (validation set not published yet) | ELISA  (Abbott Laboratories, USA) | ***CRC vs controls:***  AUC = 0.55 | NA | NA | CEA  (AUC=0.60) |
| Tissue inhibitor of Metalloprotease 2 (TIMP-2) | Yes | Groblewska (2010)  (Poland) | Prospective recruitment of CRC and patients referred for colonoscopy in one Polish center | 91 CRC, 28 adenomas, 91 HC | No training or validation set | ELISA  (R&D Systems, England) | ***CRC vs controls:***  AUC = 0.71 | NA | NA | CEA  (AUC=0.85)  CA19-9 (AUC=0.68) |
| Matrix Metalloprotéinase 1 (MMP-1) | Yes | Atwa  (2020)  (Egypt) | Prospective recruitment of patients addressed for colonoscopy in one Egyptian center | 120 CRC (56 early, 64 late), 75 benigns lesion, 56 HC | No training or validation set | ELISA | ***CRC vs controls (benign and HC):***  AUC = 0.84  Se = 75.0%  Sp = 82.5% | NA | NA | NA |
| Matrix Metalloprotéinase 2 (MMP-2) | Yes | Groblewska (2010)  (Poland) | Prospective recruitment of CRC and patients referred for colonoscopy in one Polish center | 91 CRC, 28 adenomas, 91 HC | No training or validation set | ELISA  (R&D Systems, England) | ***CRC vs controls:***  AUC = 0.69 | NA | NA | CEA  (AUC=0.85)  CA19-9 (AUC=0.68) |
| EGF Containing Fibulin Extracellular Matrix Protein 2 (EFEMP2)  (also known as Fibulin 4) | No | Yao  (2012)  (China) | Random selection of patients referred in a Chinese center. | 122 CRC, 14 adenomas, 79 HC | No training or validation set | ELISA (KPL, Inc.,  Gaithersburg, MD) | **CRC vs HC:**  AUC = 0.92  Cut-off = 1.90  Se = 82.8%  Sp = 93.7% | NA | NA | CEA  (AUC=0.73) |
| Matrix Metalloprotéinase 9 (MMP-9) | Yes | Mroczko  (2010)  (Poland) | Case-control study | 75 CRC, 35 adenomas, 70 HC | No training or validation set | ELISA  (R&D Systems, England) | ***CRC vs HC:***  AUC = 0.56 | NA | NA | CEA  (AUC=0.83)  CA19-9 (AUC=0.56) |
|  | Yes | Wilson  (2012)  (England) | Prospective recruitment of British patients (use of survey on gastro-intestinal symptoms). | 46 AN (3 CRC + 43 AA), 165 low-risk polyps, 12 IBD, 525 HC | No training or validation set | ELISA  (R&D Systems, Abingdon, UK) | ***AN vs others:***  AUC = 0.77  Se = 79.0%  Sp= 70.0% | NA | NA | NA |
|  | Yes | Otero-Estevez (2015)  (Spain) | Prospective recruitment of asymptomatic patients with at least one risk factor of CRC | 4 CRC, 53 AA, 121 NAA, 338 controls | No training or validation set | ELISA  (eBioscience, Austria) | ***AN vs controls:***  AUC = 0.68 (0.64-0.72)  Se = 19.3%  Sp = 90.0% | NA | ***AA vs controls:***  AUC = 0.69 (0.65-0.73)  Se = 22.6%  Sp = 90.0% | FIT at 100 ng/mL  (AUC =0.74) |
|  | Yes | Gimeno-Garcia  (2016)  (Spain) | Prospective recruitment of patients referred for colonoscopy in one Spanish center | 25 CRC, 25 NAA, 25 AA, 75 HC | No training or validation set | Fluorokine Multianalyte Profiling kits (R&D Systems, USA)  And the Luminex 200 system (Luminex corp, USA) | ***AN vs controls (NAA and HC):***  AUC = 0.80  Cut-off: 204 ng/mL  Se = 80.0%  Sp = 67.0% | NA | NA | NA |
| Collagen type X alpha1 (COL10A1) | No | Solé  (2014)  (Spain) | Case-control study of Spanish patients | 80 CRC, 23 adenomas, 77 HC | Discovery set in tissue, validation set in tissue and serum samples. | ELISA  (Life Sciences Inc and R&D Systems). | ***CRC + adenomas vs controls****:*  AUC = 0.76  Cut-off: 280 ng/mL  Se = 63.0%  Sp = 85.0% | NA | NA | NA |
| Tenascin-c (TNC) | Yes | Zhou  (2019)  (China) | Case-control study of Chinese patients | 258 CRC, 99 benign lesion (IBD and polyps), 103 HC | No training or validation set | ELISA  (Cusabio, China) | ***CRC vs HC:***  AUC = 0.77 (0.73-0.81)  Cut-off: 3.87 ng/mL  Se = 57.0%  Sp = 90.1% | NA | NA | CEA  (AUC=0.74)  CA19-9  (AUC=0.66) |
| Fibulin-1 (FBLN1) | No | Watany  (2018)  (Egypt) | Case-control study of Egyptian patients | 49 CRC, 26 benign polyps, 45 HC | No training or validation set. | ELISA  (LSBio, life Span BioScience, USA) | ***CRC vs controls:***  AUC = 0.98  Cut-off : 2.6 ng/mL  Se = 93.9%  Sp = 90.1% | NA | NA | CEA  (AUC=0.96) |
| Cysteine-rich angiogenic protein 61 (CYR61) | Yes | Song  (2016)  (China) | Case-control study of Chinese patients | 137 CRC, 73 adenomas, 172 HC. | No training or validation set. | ELISA  (R&D Systems) | ***CRC vs controls:***  AUC = 0.94  Cut-off: 92.0 pg/mL  Se = 83.0%  Sp = 97.0% | NA | NA | CEA  (AUC=0.77)  CA19-9  (AUC=0.67) |
| Circulating Cytokeratin 19 fragment (CyFra 21-1) | Yes | Thomas  (2015)  (England) | A nested case-Control study within the UK collaborative trial for ovarian cancer screening, involving asymptomatic British women with blood sample collected before diagnosis. | 40 CRC (20 early and 20 late stage), 20 benign colorectal neoplasms, 40 matched controls | No training or validation set. | Cobas immunoassays  and platform (Roche Diagnostics, Burgess Hill, UK) | **CRC vs controls (0-1 years before diagnosis)**:  Cut-off: 2 ng/mL  Se = 14.6%  Sp = 90.0% | NA | No difference | CEA (0-1 years before diagnosis):  (AUC=0.74) |
|  | Yes | Kleif  (2022)  (Denmark) | Prospective recruitment of asymptomatic Danish patients with a positive FIT | 242 CRC, 2064 adenomas, 1742 HC | Training set (validation set not published yet) | ELISA  (Abbott Laboratories, USA) | ***CRC vs controls:***  AUC = 0.58 | NA | NA | CEA  (AUC=0.60) |
|  | Yes | Ke  (2023)  (China) | Case-control study of Chinese patients | 74 CRC, 74 precancerous, 61 HC | Training set and validation set | Electrochemical Luminescence Immunoassay (Roche, Switzerland) | ***CRC and precancerours lesion vs HC:***  AUC = 0.68 (0.60-0.76) | NA | NA | CEA  (AUC=0.79)  CA19-9  (AUC=0.77) |
| Cytokeratin-1 (CK1) | Yes | Attallah (2018)  (Egypt) | Case-control study of Egyptian patients | 150 CRC,  50 benign growths,  35 HC | No training or validation set | ELISA  (ABC Diagnostics, Egypt) | ***CRC vs benign growths:***  AUC = 0.75 (0.68-0.83)  Cut off: 0.35 µg/mL  Se = 70.8%  Sp = 73.3% | ***Early vs Late stage:***  AUC = 0.63  (0.53-0.73)  Cut off : 2.75 µg/mL  Se = 70.6%  Sp = 72.0% | NA | CEA  (AUC=0.58)  CA19-9  (AUC=0.60) |
| Cytokeratin-20  (CK20) | No | Liu  (2021)  (China) | Case-control study of Chinese patients | 120 CRC, 60 adenomas, 63 HC | Training set and validation set | ELISA (Cusabio, China) | ***CRC vs others:***  Se = 36.7%  Sp = 88.3% | NA | NA | NA |
| ITGB4 (Integrin B4) or CD104 | Yes | Jiang (2021)  (China) | Prospective recruitment of Chinese patients | 98 CRC, 532 adenomas, 1099 HC | Discovery set and validation set | ELISA  (Biorbyt) | ***CRC vs controls (adenomas and HC) :***  AUC =0.74  Cut-off: 0.70 ng/mL  Se = 79.6%  Sp = 53.2% | NA | ***Adenomas vs HC :***  AUC = 0.62  Se = 58.5%  Sp = 60.9% | CEA  (AUC=0.70) |
| Serum β-catenin | No | Li  (2019)  (China) | Case-control study of Chinese patients, with prospective recruitment | 160 CRC, 103 polyps, 64 HC | No training set or validation set | ELISA  (Cusabio, China) | ***CRC vs controls:***  AUC = 0.80 | NA | ***Polyps vs controls:***  AUC = 0.74  Se = 86.4%  Sp = 51.6% | CEA  (AUC=0.67) |
| Serum E-cadherin (also known as cadherin-1) | No | Weiss  (2011)  (Germany) | Case control study with prospective recruitment of German patients admitted in an Internal Medicine department. | 59 CRC, 36 Familial Adenomatous Polyposis, 20 polyps, 18 IBD, 45 controls | No training or validation set. | ELISA (Takara, Japan) | ***CRC (stage II-IV) vs controls:***  Cut-off: 4,75 µg/L  Se = 52.0%  Sp = 81.0% | NA | NA | CEA  (no AUC,  Se = 60.0%,  Sp = 94.0%) |
| Adiponectin | No | Zekri  (2015)  (Egypt) | Prospective recruitment of egyptian patients referred for colonoscopy. | 34 CRC, 27 colonic polyps, 24 IBD, 29 HC. | No training or validation set | ELISA (RayBiotech, USA) | ***CRC vs IBD:***  AUC = 0.85  Cut-off = 3940  Se = 76.7%  Sp = 76.0% | NA | NA | NA |
| Visfatin (also known as Nicotinamide Phosphoribosyl-  Transferase) | No | Zekri  (2015)  (Egypt) | Prospective recruitment of egyptian patients referred for colonoscopy. | 34 CRC, 27 colonic polyps (CP), 24 IBD, 29 HC. | No training or validation set | ELISA (RayBiotech, USA) | ***CRC vs polyps:***  AUC = 0.70  Cut-off = 2.4  Se = 65.5%  Sp = 66.7% | NA | NA | NA |
| Nicotinamide N-methyltransferase (NNMT) | Yes | Bünger (2012)  (Germany) | Retrospective random selection from a German biological collection | 164 CRC, 34 adenomas (12 NAA and 22 AA), 119 HC | Training set and validation set | Development of a nine-biomarkers biochip array divided in two multiplex biochips (CRCSI et II) | ***CRC vs controls:***  AUC = 0.52 (0.46-0.58) | NA | NA | CEA  (AUC=0.69) |
| Serum Netrin-1 | Yes | Li  (2020)  (China) | Case-control study from a Chinese biological collection | 50 CRC, 50 AA, 150 HC | Clinical set and a Screening set | ELISA  (SEB827Hu, Cloud-Clone Corp, USA) | ***CRC vs controls:***  AUC = 0.76 (0.68-0.84)  Se = 90.0%  Sp = 46.0% | NA | NA | NA |
| Serotonin | No | Dowling (2015)  (Ireland) | Case-control study | 40 CRC, 20 benign lesions, 20 HC | Discovery set and validation set | ELISA  (Eno Life Sciences, USA) | ***CRC (stage I-II) vs controls:***  AUC = 0.57 | NA | NA | NA |
| Brain-derived neurotrophic factor (BDNF) (also known as abrineurin) | Yes | Wang  (2021)  (China) | Case-control study including Chinese patients | 81 CRC, 31 adenomas, 61 HC | No training or validation set | ELISA (Abcam,  Cambridge, United Kingdom) | ***CRC vs adenomas:***  AUC = 0.72  Cut-off: 7.36 ng/mL  Se = 60.5%  Sp = 80.6% | NA | NA | CEA (CRC vs adenomas: AUC=0.73) |
| Neurotensin (NTS) | Yes | Qiu  (2019)  (United Kingdom) | Prospective recruitment of asymptomatic British patients referred for a screening colonoscopy. | 20 CRC, 26 adenomas, 7 HP, 27 others colonic diseases, 84 HC | No training or validation set (use of a tissue-bank independent analysis, but with another cut-off) | ELISA (catalogue number ABX152510, Abbexa Ltd., Cambridge, UK) | ***CRC and polyps (adenomas and hyperplastic) vs controls:***  AUC = 0.63  Cut-off: 550 pg/mL  Se = 60.4%  Sp = 71.6% | NA | ***Adenomas vs controls****:*  AUC = 0.67  Cut-off: 534 pg/mL  Se = 69.7%  Sp = 68.6% | NA |
| Gamma enolase (also known as enolase-2 or neuron specific enolase : NSE) | No | Dowling (2015)  (Ireland) | Case-control study | 40 CRC, 20 benign lesions, 20 HC | Discovery set and validation set | ELISA | ***CRC (stage I-II) vs controls:***  AUC = 0.55 | NA | NA | NA |
| CD24 | Yes | Kraus (2015)  (Israel) | Prospective recruitment of Israelian patients referred for colonoscopy, some of them with an already known CRC | 35 CRC, 35 adenomas, 77 HC | Training set and validation set | Western Blot assessing CD24 expression in peripheral blood leukocytes (PBLs) | ***CRC vs controls:***  Se = 80.0%  Sp =75.3% | NA | **Adenomas vs controls:**  Se = 89.2%  Sp =71.4% | NA |
| CD26 (also known as dipeptidyl peptidase-4 or adenosine deaminase complexing protein-2) | Yes | De Chiara  (2010)  (Spain) | Prospective recruitment of patients referred for colonoscopy (for any reason) | 33 CRC, 108 polyps (adenoma or hyperplastic),  90 other bowel diseases, 68 HC | Validation study (to confirm prior results, with known cut-off) | ELISA  (Bender Medsystems, Austria) | ***CRC vs controls:***  AUC = 0.81 (0.72-0.88)  Cut-off: 460 ng/mL  Se = 81.8%  Sp = 79.4% | NA | **AN vs controls:**  Cut-off : 460 ng/mL  Se = 58.0%  Sp = 75.5% | NA |
|  | Yes | Bünger (2012)  (Germany) | Retrospective random selection from a German biological collection | 164 CRC, 34 adenomas (12 NAA and 22 AA), 119 healthy controls | Training set and validation set | Development of a nine-biomarkers biochip array divided in two multiplex biochips (CRCSI et II) | ***CRC vs controls:***  AUC = 0.64 (0.58-0.70) | NA | NA | CEA  (AUC=0.69) |
|  | Yes. | Tao  (2012)  (Germany) | Retrospective random recruitment from two German biological collections | 179 CRC, 193 AA, 225 HC | No training or validation set | ELISA, Bender Medsystems (Vienna, Austria) | ***CRC vs controls:***  AUC = 0.61 (0.56-0.66)  Cut-off: 133.5 ng/mL  Se = 0.0 %  Sp = 97.2% | NA | ***AA vs controls:***  AUC = 0.54 (0.49-0.59)  Se = 4.4%  Sp = 97.2% | FIT  AUC = 0.90  Cut-off: 24 ng/mL  Se = 65.7%  Sp = 97.7% |
|  | Yes | Otero-Estevez (2014)  (Spain) | Prospective recruitment of asymptomatic patients with at least one risk factor of CRC | 4 CRC, 53 AA, 121 NAA, 338 controls | No training or validation set | ELISA  (eBioscience, Austria) | ***AN vs controls:***  AUC = 0.75 (0.71-0.79)  Cut-off: 330 ng/mL  Se = 42.1%  Sp = 90.0% | NA | **AA vs controls:**  Cut-off : 330 ng/mL  Se = 39.6%  Sp = 90.0% | FIT at 100 ng/mL  (AUC =0.72) |
|  | Yes | De Chiara  (2022)  (Spain) | Prospective recruitment of patients referred for colonoscopy (for any reason) | 249 CRC, 372 AA, 452 NAA, 46 polyps, 240 others, 344 HC | No training or validation set | ELISA, eBioscience (Vienna, Austria) | ***CRC vs controls :***  Se = 71.4%  Cut-off : 440 ng/mL | NA | ***AA vs controls:***  Se = 71.6%  Cut-off : 440 ng/mL | FIT at 100 ng/mL  (no direct comparison) |
| DDP4 enzyme activity (of the CD26 protein) | Yes | De Chiara  (2022)  (Spain) | Prospective recruitment of patients referred for colonoscopy (for any reason) | 249 CRC, 372 AA, 452 NAA, 46 polyps, 240 others, 344 HC | No training or validation set | ELISA, Merck Sigma Aldrich  (Burlington, USA) | ***CRC vs controls :***  Se = 88.9%  Cut-off : 42 mUI/mL | NA | ***AA vs controls:***  Se = 75.9%  Cut-off : 42 mUI/mL | FIT at 100 ng/mL  (no direct comparison) |
| M2-pyruvate kinase (M2-PK) (also known as tumor M2-PK, or thyroid-hormone binding protein) | Yes | Meng  (2012)  (China) | Prospective recruitment of Chinese patients addressed for colonoscopy | 93 CRC, 41 AA, 137 NAA, 47 other polyps, 7 IBD, 158 HC | No training or validation set | ELISA  (USCN Life Science and Technology, USA) | ***CRC vs HC :***  AUC = 0.89 (0.84-0.94)  Cut-off: 2 UI/mL  Se = 100.0%  Sp = 40.5% | NA | ***AA vs controls :***  AUC = 0.81 (0.74-0.86)  Cut-off: 2 UI/mL  Se = 95.1%  Sp = 40.5% | CEA  (AUC=0.70) |
|  | No | Dowling (2015)  (Ireland) | Case-control study | 40 CRC, 20 benign lesions, 20 HC | Discovery set and validation set | ELISA  (ScheBo Biotech AG, Germany) | ***CRC (stage I-II) vs controls:***  AUC = 0.60 | NA | NA | NA |
|  | Yes | Rigi  (2020)  (Iran) | Prospective recruitment of Iranian patients referred for screening colonoscopy in 2 centers. | 56 CRC, 53 adenomas, 69 controls. | Training set (study made to determine an optimal cut-off value) | ELISA (Giessen,  Germany) | ***CRC vs controls:***  AUC = 0.98  Cut-off: 25 U/mL  Se = 90.9%  Sp = 91.3% | NA | ***Adenomas vs controls****:*  AUC = 0.95  Cut-off: 19 U/mL  Se = 96.3%  Sp = 85.5% | NA |
| 14-3-3 proteins | No | Dowling (2015)  (Ireland) | Case-control study | 40 CRC, 20 benign lesions, 20 HC | Discovery set and validation set | ELISA  (Cusabio Biotech, China) | ***CRC (stage I-II) vs controls:***  AUC = 0.82 | NA | NA | NA |
| Receptor Binding Cancer Antigen expressed on SiSo cells (RCAS1) | Yes | Han  (2014)  (China) | Case-control study of Chinese patients | 113 CRC, 30 adenomas, 31 HC | No training or validation set | ELISA (Cusabio Biotech Co, China) | ***CRC vs controls (adenoma and HC):***  AUC = 0.87 (0.81-0.93)  Cut-off: 2.8 U/mL  Se = 82.1%  Sp = 88.5% | NA | NA | CEA  (AUC=0.78) |
| Serum Synaptophysin like 1 (sSYPL1) | No | Liu  (2020)  (China) | Case-control study of Chinese patients | 151 CRC, 73 adenomas, 89 HC | No training set or validation set | ELISA  (MyBioSource, USA) | ***CRC vs controls:***  AUC = 0.95 (0.92-0.97)  Cut-off: 75.8 ng/mL  Se = 86.1%  Sp = 91.0% | NA | NA | CEA  (AUC=0.65)  CA19-9 (AUC=0.57) |
| Cystatin-4 (CST4)  (also known as cystatin S) | Yes | Cai  (2022)  (China) | Case-control study from a Chinese retrospective cohort | 96 early CRC, 96 benign colorectal lesion, 96 HC | Training set and validation set | ELISA  (Shanghai Liangrun Bio-pharmaceutical Technology Co., China, CFDA 20173403280) | ***CRC vs controls:***  AUC = 0.93 (0.89-0.98)  Se = 53.1%  Sp = 96.9% | NA | NA | CEA  CA19-9 |
|  | Yes | Huang  (2023)  (China) | Retrospective analysis from a cohort of Chinese patients referred for colonoscopy. | 101 CRC, 156 benign colorectal lesion, 34 HC | No training or validation set. | ELISA (Shanghai Liangrun Bio-pharmaceutical Technology Co., China) | ***CRC vs controls****:*  AUC = 0,77 | NA | NA | CEA  (AUC=0,77)  CA19-9 (AUC=0,62) |
| Trimethyl Histone H4 lysine 20 (H4K20me3) | Yes | Özgür  (2019)  (Turkey) | Prospective recruitment of symptom or asymptomaticTurkish patients referred for colonoscopy | 40 CRCs, 31 adenomas, 79 HC, 34 non neoplastic findings | No training or validation set | ELISA-like measurement Epiquick Global Tri-mthyl Histone Quantification kit (Epigentk, USA) | ***CRC vs HC:***  AUC = 0.61 | NA | NA | NA |
| Histone H4 | No | Özgür  (2019)  (Turkey) | Prospective recruitment of symptom or asymptomatic Turkish patients referred for colonoscopy | 40 CRCs, 31 adenomas, 79 HC, 34 non neoplastic findings | No training or validation set | ELISA-like measurement Epiquick Total Histone H4 Quantification kit (Epigentk, USA) | ***CRC vs HC:***  AUC = 0.79  Se = 50%  Sp = 91% | NA | NA | NA |
| Carbonic anhydrase 11-19 (CA11-19) | No | Overholt (2015)  (USA) | Case control study with prospective recruitments of US CRC patients or controls referred for colonoscopy | 134 CRC, 39 adenomas, 34 HP, 151 other benign diseases, 90 HC | Validation set with a previously described cut-off | ELISA | ***CRC vs HC:***  PPV = 0.98 at cut-off 6.5 ng/mL | NA | ***Adenoma vs HC:***  PPV = 0.40 at cut-off 6.5 ng/mL | NA |
| Carbohydrate anhydrase 72-4 (CA72-4) | Yes | Ke  (2023)  (China) | Case-control study of Chinese patients | 74 CRC, 74 precancerous, 61 HC | Training set and validation set | Electrochemical Luminescence Immunoassay (Roche, Switzerland) | ***CRC and precancerours lesion vs HC:***  AUC = 0.68 (0.60-0.75) | NA | NA | CEA  (AUC=0.79)  CA19-9  (AUC=0.77) |
| Carbohydrate anhydrase 125 (CA125) | Yes | Ke  (2023)  (China) | Case-control study of Chinese patients | 74 CRC, 74 precancerous, 61 HC | Training set and validation set | Electrochemical Luminescence Immunoassay (Roche, Switzerland) | ***CRC and precancerours lesion vs HC:***  AUC = 0.58 (0.49-0.66) | NA | NA | CEA  (AUC=0.79)  CA19-9  (AUC=0.77) |
| Carbohydrate anhydrase 242 (CA242) | Yes | Ke  (2023)  (China) | Case-control study of Chinese patients | 74 CRC, 74 precancerous, 61 HC | Training set and validation set | Electrochemical Luminescence Immunoassay (Roche, Switzerland) | ***CRC and precancerours lesion vs HC:***  AUC = 0.63 (0.54-0.71) | NA | NA | CEA  (AUC=0.79)  CA19-9  (AUC=0.77) |
| Nucleoside Diphosphate Kinase A (NDKA) | Yes | Otero-Estevez  (2016)  (Spain) | Prospective cohort of asymptomatic patients with at least 1 risk factor of CRC  referred for colonoscopy | 4 CRC, 53 AA, 120 NAA, 334 controls | No training or validation set | ELISA  (Cusabio Biotech, China) | ***AN vs controls :***  AUC = 0.61 (0.56-0.65)  Cut-off: 95 pg/mL  Se = 17.5%  Sp = 95.2% | NA | **AA vs controls:**  AUC = 0.60 (0.56-0.65)  Cut-off: 95 pg/mL  Se = 17.0%  Sp = 95.2% | NA |
| Serine/threonine kinase-31 (STK31) | Yes | Watany  (2018)  (Egypt) | Case-control study of Egyptian patients | 49 CRC, 26 benign polyps, 45 HC | No training or validation set. | ELISA (Wuhan Fine Biotech Co., China) | ***CRC vs controls:***  AUC = 0,96  Cut-off: 8,3 ng/mL  Se = 95.9%  Sp = 93.0% | NA | NA | CEA  (AUC=0.96) |
| Benzoic acid | No | Uchiyama  (2017)  (Japan) | Case-control study of Japanese patients. | 56 CRC, 59 adenomas, 60 HC. | Discovery set | CE-TOFMS analysis | ***CRC vs others (adenomas+HC):***  AUC = 0.89  Cut-off: 0,000355  Se = 89.0%  Sp = 82.0% | ***Stage I vs controls:***  AUC = 0.98  Se = 100.0%  Sp = 98.0% | ***Adenomas vs HC****:*  AUC = 0.92  Cut-off: 0,000501  Se = 88.0%  Sp = 85.0% | NA |
| Mucin-1  (MUC1) | Yes | Attallah (2018)  (Egypt) | Case-control study | 150 CRC,  50 benign growths,  35 HC | No training or validation set | ELISA  (ABC Diagnostics, Egypt) | ***CRC vs benign growth:***  AUC = 0.73 (0.64-0.82)  Cut off: 2 µg/mL  Se = 74.2%  Sp = 76.0% | ***Early vs Late stage:***  AUC = 0.67 (0.58-0.7)  Cut off : 2.17 µg/mL  Se = 72.1%  Sp = 75.4% | NA | CEA  (AUC=0.58)  CA19-9  (AUC=0.60) |
| Trefoil Factor Protein 3 (TFF3) | Yes | Li  (2017)  (China) | Case-control study of Chinese patients | 127 CRC, 35 polyps, 42 HC | No training set or validation set | ELISA (SEB656Hu22, China) | ***CRC vs controls:***  AUC = 0.89 (0.85-0.93)  Cut-off: 5.6 ng/mL  Se = 74.2%  Sp = 94.8% | NA | NA | CEA  (AUC=0.72) |
| IgG autoantibodies against p53 | Yes | Chen  (2016)  (Germany) | Retrospective random selection from a German biological collection | 49 CRC, 128 adenomas (99 AA, 29 NAA), 100 HC | Training set and validation set | Multiplex bead-based serological assays | ***CRC vs controls:***  Se = 8% (3-19)  Sp = 100% (96-100) | ***Early stage vs controls:***  Se = 7% (2-23)  Sp = 100% (96-100) | ***AA vs controls:***  Se = 1% (0-6)  Sp = 100% (96-100) | NA |
|  | Yes | Fitzgerald (2019)  (Ireland) | Recruitment of symptomatic Irish patients referred for colonoscopy | 24 CRC, 53 adenomas, 37 HC | No training set or validation set | Multiplex indirect ELISA | ***CRC vs controls:***  Se = 12.5%  Sp = 97.3% | NA | ***Adenomas vs controls:***  No difference | NA |
|  | Yes | Chen  (2020)  (China) | Case control study | 301 CRC, 130 benign lesions (including 56 adenomas),  170 HC | Validation study (to confirm prior results) | ELISA | ***CRC vs controls:***  AUC = 0.64 (0.59-0.69)  Se = 23.6%  Sp = 96.7% | NA | NA | NA |
| IgG autoantibodies against HRAS | Yes | Chen  (2020)  (China) | Case control study | 301 CRC, 130 benign lesions (including 56 adenomas),  170 HC | Validation study (to confirm prior results) | ELISA | ***CRC vs controls:***  AUC = 0.63 (0.58-0.68)  Se = 18.5%  Sp = 97.0% | NA | NA | NA |
| IgG autoantibodies against NSG1 | Yes | Chen  (2020)  (China) | Case control study | 301 CRC, 130 benign lesions (including 56 adenomas),  170 HC | Validation study (to confirm prior results) | ELISA | ***CRC vs controls:***  AUC = 0.65 (0.60-0.70)  Se = 20.4%  Sp = 95.3% | NA | NA | NA |
| IgA autoantibodies against TIFgamma | Yes | Chen  (2020)  (China) | Case control study | 301 CRC, 130 benign lesions (including 56 adenomas),  170 HC | Validation study (to confirm prior results) | ELISA | ***CRC vs controls:***  AUC = 0.72 (0.67-0.76)  Se = 25.5%  Sp = 96.7% | NA | NA | NA |
| Auto antibodies against MAGE4 | Yes | Chen  (2016)  (Germany) | Retrospective random selection from a German biological collection | 49 CRC, 128 adenomas (99 AA, 29 NAA), 100 HC | Training set and validation set | Multiplex bead-based serological assays | ***CRC vs controls:***  Se = 6% (2-17)  Sp = 96% (90-98) | ***Early stage vs controls:***  Se = 11% (4-28)  Sp = 96% (90-98) | ***AA vs controls:***  Se = 12% (7-20)  Sp = 96% (90-98) | NA |

CRC = colorectal cancer, AN = advanced neoplasia, NAA = non advanced adenomas, AA = advanced adenoma, HP = hyperplastic polyps, IBD = inflammatory bowel disease, HC = healthy controls, FIT = fecal immunochemical test, AUC = area under the curve, Se = sensitivity, Sp = specificity

**Table S2: Combination of serum protein biomarkers for detecting colorectal cancer or adenoma**

| **Biomarkers** | **Role in carcino-genesis** | **Study**  **(year)**  **(country)** | **Design** | **Sample size** | **Statistical validation** | **Combination method** | **Technique of measure** | **CRC detection (vs control)** | **Comparison early vs late CRC** | **Adenoma detection**  **(vs control)** |
| --- | --- | --- | --- | --- | --- | --- | --- | --- | --- | --- |
| sHLA-G + CEA | Yes | Zhu  (2010)  (China) | Prospective recruitment of Chinese patients referred for CRC surgery or colonoscopy | 144 CRC, 65 adenomas, 72 HP, 57 IBD, 60 HC | No training or validation set | NP | ELISA  (Exbio, Czech Republic) | ***CRC vs others:***  AUC = 0.87 (0.84-0.91)  Cut-off: 88.6 U/mL for sHLA-G 5 ng/mL for CEA | NA | NA |
| CEA + CA 19-9 | Yes | Zhang  (2015)  (China) | Retrospective recruitment of patients referred for CRC surgery or patients with benign colorectal disease | 138 CRC, 111 benign disease group (24, adenomas, 41 HP, 46 others) | No training or validation set | Logistic regression | Electrochemi-luminescence analyzer Elecsys 2010 | ***CRC vs others****:* AUC = 0.80 (0.74-0.85)  Cut-off:  3.36 ng/mL for CEA and  23.9 U/mL for CA 19-9 Se = 71.7%  Sp = 82.9% | NA | NA |
| Trefoil Factor Family 3 (TFF3) + CEA | Yes | Xie  (2017)  (China) | Case-control study with non-pretreated CRC, and patients with polyps and HC. | Training set: 214 CRC (82 early, 132 late), 198 adenomas, 115 HC  Validation set: 132 CRC (56 early, 76 late), 104 adenomas, 107 HC. | Training and validation set | Logistic regression | ELISA (Elabscience Biotechnology Co., Ltd. (Wuhan, China)) | ***CRC vs HC:***  AUC = 0.94  Cut-off: 0.6  Se = 89.4%  Sp = 87.9% | NA | NA |
| CEA + anti-p53 auto-antibody | Yes | Werner  (2016)  (Germany) | Retrospective recruitment from a German biological collection | 36 CRC, 4 carcinoma in situ, 420 AA, 1200 HC | Validation study (precedent study with 6 marker-panel) | Logistic regression | Sandwich assays based on the streptavidin-biotin technology (ELISA) | ***CRC vs HC:***  AUC = 0.85  Sp = 90.0% Se = 58.0% | NA | ***AA vs HC:***  AUC = 0.56 |
| BDNF + CEA | Yes | Wang  (2021)  (China) | Case-control study of Chinese patients | 81 CRC, 31 adenomas, 61 HC | No training or validation set | NA | ELISA  (Abcam,  Cambridge, United Kingdom) | ***CRC vs adenomas:***  AUC = 0.82  (0.74-0.91)  Cut-off: 7.36 ng/mL for BDNF,  4.59 ng/mL for CEA  Se = 85.2%  Sp = 67.7% | NA | NA |
| Microtubule Associated Protein RP/EB Family Member 1 (MAPRE1) + CEA | Yes | Taguchi  (2015)  (USA) | Case-control study from an American biological collection | 30 early-stage CRC, 30 late-stage CRC, 60 adenomas, 60 HC | Validation study | And-or combination | High-density antibodies arrays + ELISA  (R&D  Systems) | ***CRC vs controls:***  AUC = 0.78  Se = 35.0%  Sp = 95.0% | ***Early CRC vs controls:***  AUC = 0.79  Se = 40.0%  Sp = 95.0% | ***Adenoma vs controls:***  AUC = 0.73  Se = 16.7%  Sp = 95.0% |
| Microtubule Associated Protein RP/EB Family Member 1 (MAPRE1) + CEA + AK1 | Yes | Taguchi  (2015)  (USA) | Case-control study from an American biological collection | 30 early-stage CRC, 30 late-stage CRC, 60 adenomas, 60 HC | Validation study. | And-or combination | High-density antibodies arrays + (R&D  Systems) | ***CRC vs controls:***  Se = 43.3%  Sp = 95.0% | ***Early CRC vs controls:***  Se = 46.7%  Sp = 95.0% | ***Adenoma vs controls:***  Se = 35.0%  Sp = 95.0% |
| Fribinogen to prealbumin ratio (FPR) + CEA + CA19-9 | No | Sun  (2018)  (China) | Case-control study of Chinese patients | 455 CRC, 455 benign colorectal polyp, 455 HC | No training or validation set | Logistic regression | SYSMEX CA-7000  Machine (Fibrinogen) + OLYMPUS AU5400 (Pre-Albumin) + electro-chemiluminescence immunoassay (SIEMENS ADVIA Centaur CP, for CEA and CA 19-9) | ***CRC vs benign disease:***  AUC = 0.85  Se = 67.9%  Sp = 85.3% | NA | NA |
| Combination of 6 markers: CEA + CYFRA 21-1 + ferritin, osteopontin (OPN) + anti-p53 + seprase | NA | Wild  (2010)  (Germany) | Prospective recruitment of patients from 2 large multicenter European studies | 301 CRC (181 colon, 120 rectum), 143 AA, 266 controls | Training and validation set | LASSO logistic regression | ELISA | ***CRC vs controls:***  Sp = 95.0%  Se = 69.6% | NA | ***AA vs controls:***  Sp = 95.0%  Se = 22.7% |
| 5-marker panel: CEA + ferritin+ seprase + osteopontin + anti-p53 | Yes | Werner  (2016)  (Germany) | Retrospective recruitment from a German biological collection. | 36 CRC, 4 carcinoma in situ, 420 AA, 1200 HC | Validation study (precedent study with 6 marker-panel) | Logistic regression | Sandwich assays based on the streptavidin-biotin technology (ELISA) | ***CRC vs HC:***  AUC = 0.78  Sp = 90.0% Se = 50.0% | NA | ***AA vs HC:***  AUC = 0.56  Sp = 90.0%  Se = 16.0% |
| TIMP-1 + CEA | Yes | Christensen (2015)  (Danmark) | Retrospective analysis of a prospective biological cohort composed of sympto or asymptomatic Australian patients referred for colonoscopy | 32 CRC, 265 adenomas, 1668 controls | Study design to validate prior results  (validation study) | Logistic regression | ELISA (Abbott, Germany) | ***CRC vs controls:***  AUC = 0.75 | NA | ***Adenomas vs controls:***  AUC = 0.60 |
|  | Yes | Nielsen  (2011)  (Denmark) | Prospective recruitment of symptomatic Danish patient referred for colonoscopy | 294 CRC, 843 adenomas, 1176 non neoplasic findings, 2173 HC | No training or validation set | Logistic regression | ELISA  (Architect i2000, Abbott Laboratories, Germany) | ***CRC vs controls:***  AUC = 0.75 (0.72-0.78) | NA | NA |
| YKL-40 + CEA | Yes | Johansen (2015)  (Denmark) | Prospective recruitment of symptomatic or asymptomatic Danish patients referred for a colonoscopy | 293 CRC, 854 adenomas, 1176 other non-malignant diseases, 2173 HC | No discovery or validation set  No details concerning ROC combination | Logistic regression | ELISA  (Quidel, USA) | ***CRC vs HC:***  AUC = 0.75 | NA | NA |
|  | Yes | Ye  (2014)  (China) | Case-control study of Chinese patients | 120 CRC, 43 benign colorectal lesions, 36 HC | No discovery or validation set | NA | ELISA | ***CRC vs controls:***  Se = 82.1%  Sp = 66.7% | NA | NA |
| ITGB4 + CEA | Yes | Jiang (2021)  (China) | Prospective recruitment of Chinese patients | 98 CRC, 532 adenomas, 1099 HC | Discovery set and validation set | Logistic regression | ELISA  (Biorbyt) | ***CRC vs others (adenomas and HC):***  AUC =0.75 | NA | ***Adenomas vs HC:***  AUC = 0.62 |
| Serum β-catenin level + CEA | No | Li  (2019)  (China) | Case-control study of Chinese patients, with prospective recruitment | 160 CRC, 103 polyps, 64 HC | No training set or validation set | NA | ELISA | ***CRC vs controls:***  AUC = 0.88  Se = 81.9%  Sp = 73.4% | NA | ***Polyps vs HC:***  AUC = 0.73 |
| Serum Synaptophysin like 1 (sSYPL1) + CEA + CA19-9 | No | Liu  (2020)  (China) | Case-control study of Chinese patients | 151 CRC, 73 adenomas, 89 HC | No training set or validation set | NA | ELISA  (MyBioSource, USA) | ***CRC vs controls:***  AUC = 0.97 (0.95-0.99)  Se = 86.8%  Sp = 96.7% | NA | NA |
| S100A9 + TNC + CEA | Yes | Zhou  (2019)  (China) | Case-control study of Chinese patients | 258 CRC, 99 benign lesion (IBD and polyps), 103 HC | No training or validation set | Logistic regression | ELISA  (Cusabio, China) | ***CRC vs HC:***  AUC = 0.91 (0.88-0.93)  Se = 79.8%  Sp = 89.6% | NA | NA |
| A combination of MIC-1 + CEA + CA19-9 + CA24-2 | Yes | Dai (2021)  (China) | Prospective recruitment from a cohort of asymptomatic Chinese patients referred for a screening colonoscopy | 19 CRC, 47 precancerous lesions, 24 randomly selected HC | Non training or validation set | NA | ELISA | ***CRC vs controls:***  AUC = 0.94 (0.86-1.00)  Se = 94.1%  Sp = 87.5% | NA | ***Precancerous lesions vs HC:***  AUC = 0.80 (0.70-0.90) |
| CEA + Total cholesterol + HDL cholesterol | Yes | Pan  (2022)  (China) | Case-control study of Chinese patients | 65 CRC, 11 polyps, 51 HC | No training set or validation set | Logistic regression | ELISA | ***CRC vs controls:***  AUC = 0.91 (0.87-0.96) | NA | NA |
| A combination of 6 biomarkers: CEA + CA19-9 + CA125 + Cyfra 21-1 + CA72-4 + CA242 | Yes | Ke  (2023)  (China) | Case-control study of Chinese patients | 74 CRC, 74 precancerous, 61 HC | Training set and validation set | Prediction score constructed through an artificial neural network analysis | Electrochemical Luminescence Immunoassay (Roche, Switzerland) | ***CRC and precancerours lesion vs HC:***  AUC = 0.92  Se = 96.0%  Sp = 50.0% | NA | NA |
| Combination of p53 et p63 proteoforms | Yes | Montero-Calle  (2023)  (Spain) | Retrospective random selection from a Spanish biological collection | 31 CRC, 31 pre-malignant lesions, 48 HC | No training or validation set | NA | ECL Western blotting substrate (Thermo Fisher Scientific, Waltham,  MA, USA) | ***CRC vs HC:***  AUC = 0.87  Se = 96.8%  Sp = 64.6% | NA | ***Adenoma vs HC:***  AUC = 0.91  Se = 77.4%  Sp = 91.7% |
| A panel of 37 biomarkers (CEA + RDV + H-FABP and others) | NA | Song  (2020)  (China) | Case-control study of Chinese patients | 350 CRC, 300 polyps, 360 HC | No training set or validation set | Prediction score constructed through an artificial neural network analysis | NA | ***CRC vs HC:***  AUC = 0.99 (0.99-1.0)  Se = 97.4%  Sp = 96.7% | NA | NA |
| 5-marker panel: GDF15 + AREG + FasL + Flt3L + anti-P53 | NA | Chen  (2017)  (Germany) | Retrospective selection from a German biological collection (BLITZ) | 267 CRC, 106 AA, 225 HC | Discovery set and validation set | LASSO logistic regression | Proximity extension assays (Olink Bioscience,  Uppsala, Sweden) | **CRC vs controls :**  AUC = 0.82 (0.74-0.90)  Se = 56.4%  Sp = 90.0% | NA | **AA vs HC :**  AUC = 0.60 (0.52-0.69)  Se = 22.0%  Sp = 90.0% |
| Anti-p53 IgG + Anti-HRAS IgG + Anti TIF1gamma-IgA | Yes | Chen (2020)  (Chinese) | Case-control study of Chinese patients | 301 CRC, 130 benign lesions (including 56 adenomas),  170 HC | Validation study (to confirm prior results) | And-or combination  (positive if each autoantibody level exceed the mean +2SD of control group) | ELISA | ***CRC vs controls:***  AUC = 0.74 (0.69-0.78)  Se = 47.1%  Sp = 92.0% | NA | NA |
| A 7- autoantibodies combination with 4 IgM (anti CADM1, ICLN, SEC16 and ZNF 768) and 3 IgG (anti HMGB1, p53 and ZNF 700) | Yes | Fitzgerald (2019)  (Ireland) | Recruitment of symptomatic Irish patients referred for colonoscopy | 24 CRC, 53 adenomas, 37 HC | No training set or validation set | And-or combination  (positive if one biomarker or more is positive) | Multiplex indirect ELISA | ***CRC vs controls :***  Se = 70.8%  Sp = 86.5% | NA | ***Adenomas vs controls :***  No difference |
| A auto-antibodies combination with 9 IgG (anti CHCHD3, CTTNBP2NL, ERP44, FKBP4, MGST3, TALDO1, THSD7A, TRIM29, p53) | Yes | Garranzo-Asensio (2019)  (Spain) | Case control study from a Spanish biological collection | 32 CRC, 18 precancerous lesion, 30 controls | Training set with limited sample size and validation set | NA | ELISA-like tests | ***CRC or adenoma vs controls:***  *AUC = 0.95*  *Se = 92%*  *Sp = 90%* | NA | NA |
| Cystatin 4 (CST4) + DR-70 | No | Cai  (2022)  (China) | Case-control study from a Chinese retrospective cohort | 96 early CRC, 96 benign colorectal lesion, 96 HC | Training set and validation set | And-or combination  (positive if each autoantibody level exceed the 95^th^ percentile of control group) | ELISA | ***CRC vs controls:***  AUC = 0.94  Se = 71.9%  Sp = 89.1% | NA | NA |
| Chemokine CC ligand 20 (CCL20)+ Interleukin 17A (IL-17A) | No for CCL-20 Yes for IL-17A. | Wang  (2019)  (China) | Case-control study of Chinese patients | Training set :  112 CRC, 59 HC, 52 Colitis, 40 Adenoma  Validation set :  75 CRC, 35 HC, 20 Colitis, 35 Adenoma | A  discovery set to screen biomarkers with multiplex assay, then a training and a validation set | Logistic regression | ELISA  (R&D Systems, Minneapolis, MN, USA) | ***CRC vs controls:***  AUC = 0.98  Se = 93.3%  Sp = 93.3% | ***Early CRC vs controls****:*  AUC = 0.98  Se = 95.1%  Sp = 93.2% | NA |
| A panel of 26 biomarkers including CEA and AFP | No | Wang  (2023)  (China) | Case control study of Chinese patients | 204 CRC, 186 AA, 198 NAA, 229 HC | No training or validation set | Logistic regression  Construction of 4 machine learning models | NA | ***CRC vs HC:***  AUC = 0.94  Se = 90.2%  Sp = 91.2% | NA | ***Adenoma vs HC:***  AUC = 0.93  Se = 90.4%  Sp = 77.1% |
| CK20 + hypermethylated CLIP4 | No | Liu  (2021)  (China) | Case-control study of Chinese patients | 120 CRC, 60 adenomas, 63 HC | Training set and validation set | NA | ELISA (Cusabio, China) for CK20  Methylation specific PCR for methylation status of CLIP4 | ***CRC vs others:***  Se = 95.0%  Sp = 81.7% | NA | NA |
| Collectin-liver 1 (CL-L1) + M-ficolin + MBL-associated protein 44 (Map44) | No | Storm  (2015)  (Denmark) | 2 consecutive case-control study | 99 CRC, 196 adenomas, 696 no neoplastic finding (HC) | A discovery study (study 1) and a validation study (study 2) | Logistic regression | Time-resolved immunofluorometric  assays (TRIFMAs), sandwich immunoassays. | ***CRC vs HC****:*  AUC = 0.68  Se = 36.0%  SP = 83.0% | NA | ***Adenoma vs HC****:*  Se = 30.0%  Sp = 79.0% |
| 8 cancer-associated protein biomarkers: AFP, CA19-9, CEA, hs-CRP, CyFra21-1, Ferritin, Galectin-3, and TIMP-1 | Yes | Wilhelmsen  (2017)  (Denmark) | Prospective recruitment of Danish patients referred for colonoscopy in 7 centers | 512 CRC, 689 adenomas (399 AA, 290 NAA), 177 other cancers, 1342 non-malignant findings, 1978 HC | No training or validation set. | Logistic regression | Abbott  ARCHITECTR i2000 automated immunoassay platform utilizing a two-step dual monoclonal  Immunoassay. | ***AN vs HC****:*  AUC = 0.76  Se = 90.0% Sp = 33.0%  ***CRC vs HC:***  AUC = 0.84  Se = 90.0% Sp = 48.0% | NA | NA |
|  | Yes | Gawel (2019)  (USA) | Retrospective analysis of a prospective biological collection composed of symptomatic US patients referred for colonoscopy (the Endoscopy II Trial) | 512 CRC, 399 AA, 290 NAA, 1342 non malignant findings, 1968 HC | Training set and validation set  Use of 6 different statistical method (the most effective – the unweighted vector fusion - was reported) | 6 different methods, among them logistic regression | The Architect i2000SR immunoassay and c8000 clinical chemistry systems (Abbots Labs, Germany) | ***AN vs others (NAA) :***  AUC = 0.84  Se = 76.0%  Sp = 79.0% | NA | NA |
| A multivariable model combining CEA + hsCRP + Human Epididymis secretory protein 4 (HE4) + ferritin + Age and Sex | Yes | Kleif  (2022)  (Denmark) | Prospective recruitment of asymptomatic Danish patients with a positive FIT | 242 CRC, 2064 adenomas, 1742 HC | Training set (validation set is not published yet) | Logistic regression | ELISA  Architect i2000 automated platform  (Abbott Laboratories, USA) | ***CRC vs HC:***  AUC = 0.73 (0.69-0.77)  Se = 23%  Sp = 90%  ***CRC vs others (adenomas and HC):***  AUC = 0.70 (0.66-0.74)  Se = 18%  Sp = 90% | NA | NA |
| CEA + Transferrin Receptor-1 (TFRC) +  and Cancer Antigen 242 (CA242). | No | Thorsen  (2013) | Case-control study from a Danish biological collection | 70 CRC, 70 adenomas, 70 other colon diseases, 70 HC, 10 others cancers | Exploratory set and validation set | Logistic regression | Proximity extension assays (Olink Bioscience,  Uppsala, Sweden) | NA | ***Early CRC vs controls****:*  AUC = 0.82  Se = 53.0%  Sp = 90.0% | NA |
| A 9-marker algorithm:  AREG + CEA + Granzyme B (GZMB) + Integrin alpha V (ITGAV) + keratin type I cytoskeletal 19 (KRT19) + monocyte chemotactic protein 1 (MCP1) + osteopontin (OPN) + PON3 + transferrin receptor protein 1 (TR) | Yes | Bhardwaj (2020)  (Germany) | Retrospective random selection from a German biological cohort (BLITZ) composed with patients referred for a screening colonoscopy | 56 CRC,  101 AA, 102 HC | Training set and validation set | LASSO logistic regression | Proximity Extension Assays  (Olink) | ***CRC vs controls:***  AUC = 0.76 (0.67-0.84)  Se = 55%  Sp = 45% | NA | NA |
| A 12-marker algorithm:  AREG + CEA + GZMB + ITGAV + KRT19 + MCP1 + PON3 + TR + Mannan-binding lectin serine protease 1 (MASP1) + retinoid acid receptor responder protein 2 (RARRES2) + S100A4 + tartrate resistant acid phosphatase type 5 (TRAP) | Yes | Bhardwaj (2020)  (Germany) | Retrospective random selection from a German biological cohort (BLITZ) composed with patients referred for a screening colonoscopy | 56 CRC,  101 AA, 102 HC | Training set and validation set | LASSO logistic regression | Proximity Extension Assays  (Olink) | NA | ***Early CRC vs controls:***  AUC = 0.75 (0.62-0.87)  Se = 61%  Sp = 35% | ***AA vs controls:***  AUC = 0.58  (0.47-0.68)  Se = 28%  Sp = 24% |
| A 6-autoantibodies combination  antiTP53 + antiMAGE4 + antiIMPDH2 + antiMDM2 + antiCTAG1 + antiMTDH | Yes | Chen  (2016)  (Germany) | Retrospective random selection from a German biological collection | 49 CRC, 128 adenomas (99 AA, 29 NAA), 100 HC | Training set for fixing cut-off at 98% specificity, and validation set | And-or combination  (positive if one biomarker or more is positive) | Multiplex bead-based serological assays | ***CRC vs controls:***  Se = 24% (15-38)  Sp = 85% (77-91) | ***Early-stage vs controls:***  Se = 30% (16-48)  Sp = 85% (77-91) | ***AA vs controls:***  Se = 25% (18-35)  Sp = 85% (77-91) |
| IL8 + CEA | NA | Bünger (2012)  (Germany) | Retrospective random selection from a German biological collection | 164 CRC, 34 adenomas (12 NAA and 22 AA), 119 HC | Training set and validation set | Logistic regression | Development of a nine-biomarkers biochip array divided in two multiplex biochips (CRCSI et II) | ***CRC vs controls:***  Cut-off:  3.2 ng/mL for CEA  39.5 pg/mL for IL-8  Se = 47.0%  Sp = 86.0% | ***Early CRC vs controls:***  Se = 33% | ***Adenomas vs controls:***  Cut-off:  3.2 ng/mL for CEA  39.5 pg/mL for IL-8  Se = 18.0%  Sp = 86.0% |
| CRP + CEA | Yes | Bünger (2012)  (Germany) | Retrospective random selection from a German biological collection | 164 CRC, 34 adenomas (12 NAA and 22 AA), 119 HC | Training set and validation set  Individuals with at least one marker above the threshold was considered as positive | Logistic regression | Development of a nine-biomarkers biochip array divided in two multiplex biochips (CRCSI et II) | ***CRC vs controls:***  Cut-off:  3.2 ng/mL for CEA  14.600 ng/mL for CRP  Se = 39.0%  Sp = 86.0% | ***Early CRC vs controls:***  Se = 28% | ***Adenomas vs controls:***  Cut-off:  3.2 ng/mL for CEA  14.600 ng/mL for CRP  Se = 15.0%  Sp = 86.0% |
| BAG family molecular chaperone regulator 4 (BAG 4) + interleukin-6 receptor subunit beta (IL6ST) + von Willebrand factor (VWF) + CD44 + EGFR. | No | Rho  (2016)  (USA) | Case-control study divided into 3 parts, with the use of 3 differents cohorts. | Discovery set:  79 CRC, 79 HC Diagnostic set: 60 CRC, 60 adenomas, 60 HC  Validation set: 514 CRC, 159 low-risk findings (HP or NAA), 59 UC, 168 HC | Discovery set, diagnostic set and validation set. | Logistic regression | ProtIA spin column (Sigma Chemical CO, St Louis, Missouri, USA) to remove albumin and IgG + Modified Luminex assays | ***CRC vs HC****:*  AUC = 0.84  Se = 70.0%  Sp = 90.0% | **CRC stage I vs controls (UC, HC, polyps):**  AUC = 0.79  Se = 62.3%  Sp = 90.0%  **CRC stage II vs controls:**  AUC = 0.85  Se = 71.6%  Sp = 90.0% | NA |
| Diagnostic metabolomic signature composed of 48 metabolites | Yes | Farshidfar (2016)  (Canada) | Case control study with retrospective selection of CRC among a Canadian cohort, and prospective recruitment for adenoma and HC among patients referred for colonoscopy | 320 CRC, 31 adenomas, 254 HC | Development of a diagnostic metabolomic signature in a training set, subsequently confirmed in a validation set | NA | Gas chromatography Mass Spectrometry | ***CRC vs controls :***  AUC = 0.91 (0.87-0.96)  Se = 85% Sp = 86% | NA | ***Adenomas vs controls:***  AUC = 0.81 (0.70-0.92) |
| Lactate to citrate ratio | NA | Gu (2019)  (China) | Case control study from a Chinese biological collection | 40 CRC, 32 adenomas, 38 HC | No training or validation set | Multivariate ROC analysis in MetaboAnalyst 3.0 | H-NMR spectrometry | ***CRC vs controls:***  AUC = 0.83 | NA | ***Adenomas vs controls:***  AUC = 0.82 |
| Acetate to glycerol ratio | NA | Gu (2019)  (China) | Case control study from a Chinese biological collection | 40 CRC, 32 adenomas, 38 HC | No training or validation set | Multivariate ROC analysis in MetaboAnalyst 3.0 | H-NMR spectrometry | ***CRC vs controls:***  AUC = 0.67 | NA | ***Adenomas vs controls:***  AUC = 0.83 |
| A model composed of seven selected discrepant protein mass peak in spectrometry | NA | Xu (2015)  (China) | Case control study with Chinese patients | 53 untreated CRC, 72 pretreated CRC, 12 adenomas, 15 HC | No training or validation set | Support Vector Machine (SVM) based model | SELDI-TOF-Mass spectrometry | ***CRC vs controls (adenoma or HC):***  Se = 96.2%  Sp = 83.3% | NA | NA |
| Set of five serum peptides: PDA018, PDA052, PDA066, PDB001, PDB007 | Yes | Uchiyama  (2018)  (Japan) | Case-control study of Japanese patients | Training set: 84 CRC, 54 HC.  Validation set: 56 CRC (14 of each stage), 60 colonic polyps, 60 HC. | Validation study, with training and validation set | NA | LC-MS/MS (Waters Corporation, Milford,  MA, USA) | ***CRC vs HC:***  AUC = 0.89  Se = 82.0%  Sp = 93.0% | ***Early CRC vs controls****:*  Stage 1:  AUC = 0.78 Se = 64.3% Sp = 93.3%  Stage 2:  AUC = 0.95  Se = 92.9%  Sp = 93.3% | ***Adenomas vs HC****:*  No difference |
| A five-marker algorithm:  alpha1antitrypsin (A1AT),  apolipoprotein A1 (APOA1), haptoglobin (HP), leucine rich alpha 2 glycoprotein (LRG1) and serum paraoxonase lactonase 3 (PON3) | Yes | Bhardwaj (2020)  (Germany) | Retrospective random selection from a German biological cohort (BLITZ) composed with patients referred for a screening colonoscopy | 56 CRC,  99 AA, 99 HC | Training set and validation set | LASSO logistic regression | Liquid Chromatography Multiplex Réaction Monitoring-Mass Spectrometry  (LC/MRM-MS) | ***CRC vs controls :***  AUC = 0.79 (0.70-0.86)  Se = 68%  Sp = 46% | NA | NA |
| A three-marker algorithm:  HP + LRG1 + PON3 | Yes | Bhardwaj (2020)  (Germany) | Retrospective random selection from a German biological cohort (BLITZ) composed with patients referred for a screening colonoscopy | 56 CRC,  99 AA, 99 HC | Training set and validation set | LASSO logistic regression | Liquid Chromatography Multiplex Réaction Monitoring-Mass Spectrometry  (LC/MRM-MS) | NA | ***Early CRC vs controls:***  AUC = 0.79 (0.66-0.89)  Se = 57%  Sp = 48% | ***AA vs controls:***  AUC = 0.65  (0.56-0.73)  Se = 41%  Sp = 25% |
| A five-marker combination:  Leucin-rich alpha-2-glycoprotein1 (LRG1) + EGFR + inter-alpha tryspin inhibitor heavy chain family member 4 (ITIH4) + hemopexine + superoxide dismutase 3 (SOD3) | NA | Ivancic (2020)  (USA) | Case controle study with prospective recruitement of CRC and patients referred for colonoscopy | 47 CRC, 72 AA, 87 NAA, 53 HC | Training set and validation set | Different methods, among them logistic regression, SVM… | Liquid chromatography tandem mass-spectrometry | ***CRC vs controls (NAA+HC):***  AUC = 0.86  Se = 89%  Sp = 70% | NA | NA |
| A 13-marker combination: histidine, glycocholate, hippuric acid, malonic  acid/3-hydroxybutyrate (3HBA), glycochenodeoxycholate, leucic  acid, methionine, maleic acid, linolenic acid, hydroxyproline/  aminolevulinate, 2-aminoadipate, N-acetylglycine, and  glyceraldehyde | Yes | Zhu  (2014)  (USA) | Prospective recruitment of US patients referred for colonoscopy | 66 CRC, 76 polyps, 92 HC | Training set and validation set | Partial least-squares discriminant analysis (PLS-DA) and Monte Carlo Cross Validation (MCCV) | Liquid chromatography tandem mass-spectrometry. | **CRC vs controls (HC):**  AUC = 0.93  Se = 96.0%  Sp = 80.0% | NA | NA |
| A model composed of 15 lipid metabolites | No | Pan  (2022)  (China) | Case-control study of Chinese patients | 65 CRC, 11 polyps, 51 HC | Training set and validation set | Logistic regression | LC-MS/MS lipiodomics analysis  (The QTRAP 6500+ LC–MS/MS System, SCIEX, Framingham, USA) | ***CRC vs controls:***  AUC = 0.82 (0.72-0.91)  Se = 81.5%  Sp =81.5% | NA | NA |
| A four-peptides signature | NA | Deng  (2013)  (China) | Case-control study with Chinese patients | 67 CRC, 55 adenomas, 65 HC | Training set and validation set | NA | MALDI-TOF Mass Spectrometry | ***CRC vs controls (adenomas and HC):***  Se = 97.1% | NA | NA |
| A model composed of 49 N-glycans alterations | NA | Pan  (2021)  (China) | Case control study with Chinese patient | 163 CRC, 98 AA, 101 HC | Training set and validation set | Model constructed through machine learning, using SVM method | MALDI-Mass Spectromery (Shimadzu Corp, Japan) | ***AN (CRC+AA) vs HC:***  Accuracy = 87.0%  Se = 96.2%  Sp = 77.3% | NA | NA |
| GlycoF : combination of 4 IgG N-glycan (GP1, GP3, GP4, GP14) + CEA | Yes | Gu  (2022)  (China) | Case-control study with Chinese patients | 94 CRC, 99 AA, 59 HC | No training or validation set | Logistic regression | Ultra-performance liquid chromatography | ***CRC vs controls:***  AUC = 0.84  Se = 72.3%  ***CRC vs AA****:*  AUC = 0.85  Se = 61.6%  Sp = 87.2% | NA | NA |

**Table S3: Combination of serum protein biomarker to fecal immunochemical test for detecting colorectal cancer or adenoma**

| **Combination** | **Immunochemical test and cut-off used** | **Study**  **(year)**  **(country)** | **Design** | **Sample size** | **Statistical methodology** | **Technique of measure** | **CRC detection (vs control)** | **Comparison early vs late CRC** | **Adenoma detection**  **(vs control)** | **Comparison to other biomarkers** |
| --- | --- | --- | --- | --- | --- | --- | --- | --- | --- | --- |
| sCD26 + FIT | OC Censor ®  (Eiken Chemical, Japan)  (cut-off at 100ng Hb/mL or 20 µg/g stool) | Otero-Estevez (2014)  (Spain) | Prospective recruitment of asymptomatic patients with at least one risk factor of CRC | 4 CRC, 53 AA, 121 NAA, 338 controls | No training or validation set  Combination was considered as positive at least one of the two tests were positive, and negative if the two were negative | ELISA  (eBioscience, Austria) | NA | NA | ***AA vs controls:***  Cut-off for sCD26 : 330 ng/mL and for FIT : 100 ng/mL  Se = 62.3%  Sp = 90.0%  ***AN vs controls:***  Cut-off for sCD26: 330 ng/mL and for FIT : 100 ng/mL  Se = 64.9%  Sp = 90.0% | NA |
| Nucleoside Diphosphate Kinase A (NKDA) + FIT | OC Censor ®  (Eiken Chemical, Japan)  (cut-off at 100ng Hb/mL or 20 µg/g stool) | Otero-Estevez  (2016)  (Spain) | Prospective cohort of asymptomatic patients with at least 1 risk factor of CRC  referred for colonoscopy | 4 CRC, 53 AA, 120 NAA, 334 controls | No training or validation set | ELISA  (Cusabio Biotech, China) | NA | NA | ***AA vs controls:***  Cut-off for NKDA : 82 pg/mL and for FIT : 100 ng/mL  Se = 45.3%  Sp = 88.3%  ***AN vs controls :***  Cut-off for NKDA : 82 pg/mL and for FIT : 100 ng/mL  Se = 49.1%  Sp = 88.3% | NA |
| TIMP-1 + iFOBT | RIDASCREEN Haemoglobin®  (R-Biopharm AG, Germany)  (cut off at 2 µg/g stool) | Tao (2012) | Retrospective random recruitment from two German biological collections | 179 CRC, 193 AA, 225 HC | No training or validation set | ELISA, Bender Medsystems (Vienna, Austria) | ***CRC vs controls:***  No increase of AUC comparing to iFOBT alone | NA | ***AA vs controls:***  AUC = 0.71  Se = 21.3%  Sp = 97.7% | iFOBT alone  AUC = 0.68 |
| CRP + sCD26 + TIMP-1 + iFOBT | RIDASCREEN Haemoglobin®  (R-Biopharm AG, Germany)  (cut off at 2 µg/g stool) | Tao (2012) | Retrospective random recruitment from two German biological collections | 179 CRC, 193 AA, 225 HC | No training or validation set | ELISA, Bender Medsystems (Vienna, Austria) | ***CRC vs controls:***  No increase of AUC comparing to iFOBT alone | NA | ***AA vs controls:***  AUC = 0.73  Se = 21.9%  Sp = 97.7% | iFOBT alone  AUC = 0.68 |
| sCD26 + DDP4 enzyme activity + FIT | OC sensor®  (Eiken Pharmaceuticals, Japan)  (cut off at 100 ng Hb/mL) | De Chiara  (2022)  (Spain) | Prospective recruitment of patients referred for colonoscopy (for any reason) | 249 CRC, 372 AA, 452 NAA, 46 polyps, 240 others, 344 HC | No training or validation set | ELISA, eBioscience (Vienna, Austria)  Merck Sigma Aldrich  (Burlington, USA) | ***CRC vs controls :***  Se = 95.2% | NA | ***AA vs controls:***  Se = 80.3% | FIT at 100 ng/mL  (no direct comparison) |
| CEA + hsCRP + Ferritin + TIMP-1 + Pepsinogen-2 + HE4 = Cyfra 21-1 + Galectin-3 + B2M + FIT + age and sex | OC sensor®  (Eiken Pharmaceuticals, Japan)  (cut off at 100 ng Hb/mL) | Petersen  (2023)  (Denmark) | Recruitment of Danish patients addressed for colonoscopy after a positive FIT | 242 CRC, 2064 adenomas, 1742 HC | No training or validation set. | Chemiluminescence microparticle immunoassay (Architect i2000 and Architect c8000) | ***CRC vs non-CRC:***  AUC = 0.75 | NA | NA | FIT alone:  AUC = 0.69 |
